# Genome-Wide Search for SNP Interactions in GWAS Data: Algorithm, Feasibility, Replication Using Schizophrenia Datasets

**DOI:** 10.3389/fgene.2020.01003

**Published:** 2020-08-28

**Authors:** Kwan-Yeung Lee, Kwong-Sak Leung, Suk Ling Ma, Hon Cheong So, Dan Huang, Nelson Leung-Sang Tang, Man-Hon Wong

**Affiliations:** ^1^Department of Computer Science and Engineering, The Chinese University of Hong Kong, Hong Kong, China; ^2^Department of Psychiatry, The Chinese University of Hong Kong, Hong Kong, China; ^3^School of Biomedical Science, The Chinese University of Hong Kong, Hong Kong, China; ^4^Hong Kong Branch of CAS Center for Excellence in Animal Evolution and Genetics, School of Biomedical Sciences, The Chinese University of Hong Kong, Hong Kong, China; ^5^KIZ-CUHK Joint Laboratory of Bioresources and Molecular Research of Common Diseases, Kunming Institute of Zoology, The Chinese University of Hong Kong, Hong Kong, China; ^6^Margaret K.L. Cheung Research Centre for Management of Parkinsonism, The Chinese University of Hong Kong, Hong Kong, China; ^7^Shenzhen Research Institute, The Chinese University of Hong Kong, Shenzhen, China; ^8^Brain and Mind Institute, The Chinese University of Hong Kong, Hong Kong, China; ^9^Cytomics Ltd, Shatin, Hong Kong; ^10^Department of Chemical Pathology and Li Ka Shing Institute of Health Sciences, Faculty of Medicine, The Chinese University of Hong Kong, Hong Kong, China; ^11^Functional Genomics and Biostatistical Computing Laboratory, CUHK Shenzhen Research Institute, Shenzhen, China

**Keywords:** schizophrenia, GWAS, exhaustive search, second order SNP–SNP interaction, gene–lncRNA interactions

## Abstract

In this study, we looked for potential gene-gene interaction in susceptibility to schizophrenia by an exhaustive searching for SNP–SNP interactions in 3 GWAS datasets (phs000021:phg000013, phs000021:phg000014, phs000167) using our recently published algorithm. The search space for SNP–SNP interaction was confined to 8 biologically plausible ways of interaction under dominant-dominant or recessive-recessive modes. First, we performed our search of all pair-wise combination of 729,454 SNPs after filtering by SNP genotype quality. All possible pairwise interactions of any 2 SNPs (5 × 10^11^) were exhausted to search for significant interaction which was defined by *p*-value of chi-square tests. Nine out the top 10 interactions, protein coding genes were partnered with non-coding RNA (ncRNA) which suggested a new alternative insight into interaction biology other than the frequently sought-after protein–protein interaction. Therefore, we extended to look for replication among the top 10,000 interaction SNP pairs and high proportion of concurrent genes forming the interaction pairs were found. The results indicated that an enrichment of signals over noise was present in the top 10,000 interactions. Then, replications of SNP–SNP interaction were confirmed for 14 SNPs-pairs in both replication datasets. Biological insight was highlighted by a potential binding between FHIT (protein coding gene) and LINC00969 (lncRNA) which showed a replicable interaction between their SNPs. Both of them were reported to have expression in brain. Our study represented an early attempt of exhaustive interaction analysis of GWAS data which also yield replicated interaction and new insight into understanding of genetic interaction in schizophrenia.

## Introduction

Schizophrenia is a highly heritable disorder and it affected about 1% of the population worldwide (Sullivan et al., 2003, p. 20; Henriksen et al., 2017; Avramopoulos, 2018; Weinberger, 2019). Twins studies suggested the heritability is around 80% (Sullivan et al., 2003; Henriksen et al., 2017; Avramopoulos, 2018) and common variants contributed to up to half of the genetic risk of schizophrenia (International Schizophrenia Consortium et al., 2009; The Schizophrenia Psychiatric Genome-Wide Association Study (Gwas) Consortium, 2011). Genome-wide association studies (GWAS) identified more than 180 loci that were associated with the risk of schizophrenia (Ripke et al., 2014; Li et al., 2017; Pardiñas et al., 2018). Some of the genes were well known target for treatment such as dopamine receptor D2 (DRD2) and some new genes related to immune system were identified, which provided new target for therapy development. However, the SNPs identified by GWAS only explained a small effect on the disease risk (Manolio et al., 2009) and a large subset of SNPs associated with the disease is uncovered.

Single SNPs often have a small effect on the phenotype and they cannot account for all the genetic susceptibility of diseases. Many researchers explored various ways to re-analyse the GWAS data using approaches on top of the prevailing single SNP analysis, commonly used in GWAS analysis. For example, sub-classification of the phenotypes (Ruderfer et al., 2018), integration of omics data (Jaffe et al., 2018) and various ways of pathway or network analysis had been performed (Wang et al., 2019). On the other hand, it is recognized that SNP–SNP interaction can act as a stronger risk factor by working synergistically. Recently, the specific mode of enhancer-promoter interaction in GWAS had been pursued (Wu and Pan, 2018). A study showed some SNPs were not associated with the phenotypes of the disease when they were examined individually and they were only identified when examined in combination (Gerke et al., 2009). Our previous study on IGF1 promoter showed the interaction between a pair of SNPs and short tandem repeat (STR) resulted in the regulation on the level of circulating IGF1 (Chen et al., 2011, 2013, 2016). However, the association was not significant when individual SNP was examined. Furthermore, a recent study showed that the weak interaction of transcription factor to its promoter was able to regulate the expression of the gene (de Boer et al., 2020), further supporting SNP–SNP interaction provided synergistic effect on gene regulation. Other than SNP–SNP interaction occurring on the same gene, we and others showed SNP–SNP interaction across different genes were also important in determining the risk or severity of diseases including psoriasis (Lee et al., 2018), schizophrenia (Schrode et al., 2019), cancer (Lin et al., 2013), and obesity (Dong et al., 2017).

Genome-wide association studies is an important tool to identify SNP associating with a variety of diseases. However, only marginal effects of SNPs were detected. SNP–SNP interaction played an essential role in the pathogenesis of complex diseases (Phillips, 2008). To examine the SNP–SNP interaction in a GWAS dataset, there were over 10 billions of pairwise SNP combinations and it caused a huge demand of computational power. One approach was to limit the pairing of SNPs using specific features like genomic location in a study focused to the scope of Enhancer–Promoter interaction (Wu and Pan, 2018). With the improvement of computational power and better algorithm, it is now possible to exhaust all possible pairwise SNP combinations in a GWAS dataset to calculate the statistical significance of all possible pairwise interactions (Wan et al., 2010; Zhu et al., 2013; Lee et al., 2018). Another challenge for the detection of SNP-SNP interaction is arose from the multiple testing and interactions with weak effect size will not be detected under the stringent threshold. Exhaustive search approach is one of the major categories for detecting SNP–SNP interaction and the multi-factor dimensionality reduction (MDR) approach generates 3 × 3 genotype tables which may predict for high risk and low risk genotype. However, the SNP–SNP interaction identified might not be biologically interpretable.

We developed an algorithm which generated eight biological plausible SNP-SNP interactions (Chu et al., 2016) and identified some novel SNP-SNP interactions associating with the risk of psoriasis in our previous study (Lee et al., 2018). In this study, we utilized this algorithm to perform the exhaustive search for statistically significant 2nd order SNP–SNP interactions from our discovery dataset phs000021:phg000013. First, we found that 9 out of the top 10 SNP-SNP interactions in terms of *p*-value could be interpreted as the interactions between protein coding genes and non-coding RNA (ncRNA) genes which suggested the importance of interactions other than that of the traditional protein-protein interactions. After that, we investigated the replication among the top 10,000 SNP-SNP interactions and there was a high proportion of concurrent genes among the gene-gene interaction predicted from these SNP–SNP interaction. Therefore, there was an enrichment of signals over noise among these interactions. Finally, 9 SNP–SNP interactions were successfully replicated in both replication datasets. Among these SNP–SNP interactions, one of them could be interpreted as the interaction between FHIT (protein coding) and LINC00969 (lncRNA). Both of them were reported to have expression in brain.

## Materials and Methods

### Restricting Search Space of SNP–SNP Interaction With Biologically Plausible Genotype Interaction Patterns

**FIGURE 1:**
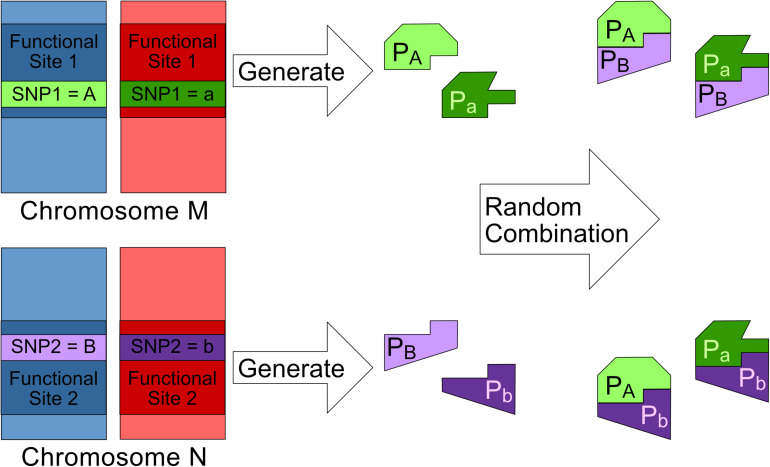
This figure shows the bio-molecule interaction mechanism behind a 2nd order SNP–SNP interaction where SNP_1_ and SNP_2_ are both having genotype (Major, Minor). Major alleles represented by upper-case letters (i.e., A, B) and minor alleles represented by lower-case letters (i.e., a, b) (Lee et al., 2018).

The distribution of different genotypes of every 2nd order SNP combination across cases and controls can be measured and visualized as a 3 × 3 genotype table. Each genotype is represented as a cell in the 3 × 3 genotype table and can be labeled as high-risk or low-risk through statistical or heuristic algorithms like multi-factor dimensionality reduction (MDR) algorithm and its derivatives (Gola et al., 2015). However, the interactions found by these algorithms may have labeling patterns which may not be explained biologically. In this paper, we have applied eight 2nd order biological plausible SNP–SNP interaction labeling patterns (Chu et al., 2016; Lee et al., 2018) for labeling genotypes as high-risk or low risk in our exhaustive search. The principles and assumptions in deriving these eight SNP–SNP interaction patterns are shown in Figure 1 and are explained below.

1. *SNP*_1_ and *SNP*_2_ are found in two different functional sites namely *Site*_1_ and *Site*_2_ respectively. (Letters “*A*” and “*B*” represent the major alleles of *SNP*_1_ and *SNP*_2_ respectively. Meanwhile, letters “a” and “b” represent the minor alleles of *SNP*_1_ and *SNP*_2_ respectively).
2. *SNP*_1_ and *SNP*_2_ can affect their respective functional sites and cause each site to generate at most two different subtypes of bio-molecules. For example, bio-molecules *p*_*A*_ is generated from *Site*_1_ with *SNP*_1_ having a major allele.
3. The bio-molecules generated from *Site*_1_ and *Site*_2_ can randomly dock with each other to form at most four different bio-molecule complexes For example, bio-molecules *p*_*A*_ and *p*_*B*_ can combine with each other to form complex *p_*A*_p_*B*_*.
4. A bio-molecule complex is considered to be associated to the genetic disease if any one of the following two conditions is satisfied:
  a. Its dominant interaction can either promote or inhibit a disease.
  b. Its recessive interaction presence can either promote or inhibit a disease.

**FIGURE 2:**
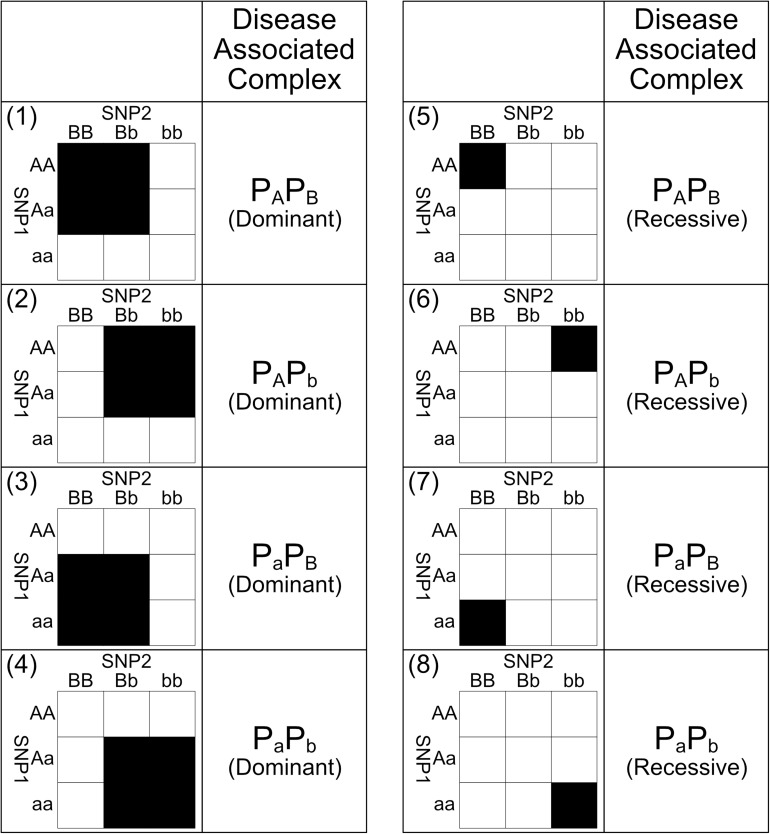
This figure shows the eight biologically plausible 2nd order genotype labeling patterns and their corresponding disease-associated complexes. Under dominant interaction patterns 1, 2, 3, and 4, their corresponding disease-associated complexes are presence in the samples carrying the black genotypes and absence in the counterpart genotypes. Meanwhile, under recessive interaction patterns 5, 6, 7, and 8, their corresponding disease-associated complexes are the only presence in the samples carrying the black genotypes and other complexes are presence in the counterpart genotypes. Among these eight interaction patterns, black genotypes and white genotypes have different risk levels caused by the difference in concentration of the disease associated bio-molecule complexes. Major alleles are represented by upper-case letters (i.e., A, B) and minor alleles are represented by lower-case letters (i.e., a, b) (Lee et al., 2018).

Those eight SNP–SNP interaction patterns are shown in Figure 2. The pattern 1 in Figure 2 is derived through the following procedure. Without the loss of generality, assuming that *p_*A*_p_*B*_* is the only bio-molecule complex associated to the genetic disease. If the dominant presence of *p_*A*_p_*B*_* can either promote or inhibit the risk of a genetic disease, samples carrying genotype *{AA, BB}*, *{AA, Bb}*, *{Aa, BB}*, and *{Aa, Bb}* obviously would have a different disease risk level comparing to other samples. Pattern 1 is hence derived after labeling these genotypes with two different colors to reflect their difference in risk level. On the other hand, if only the recessive presence of *p_*A*_p_*B*_* can either promote or inhibit a disease, samples carrying genotype *{AA, BB}* would have a different disease risk level comparing to other samples. Pattern 5 is hence derived after labeling these genotypes with two different colors to reflect their difference in risk level. Other patterns shown in Figure 2 can be also defined through a similar procedure shown above.

### Finding Statistically Significant SNP–SNP Interactions With Exhaustive Search

**FIGURE 3:**
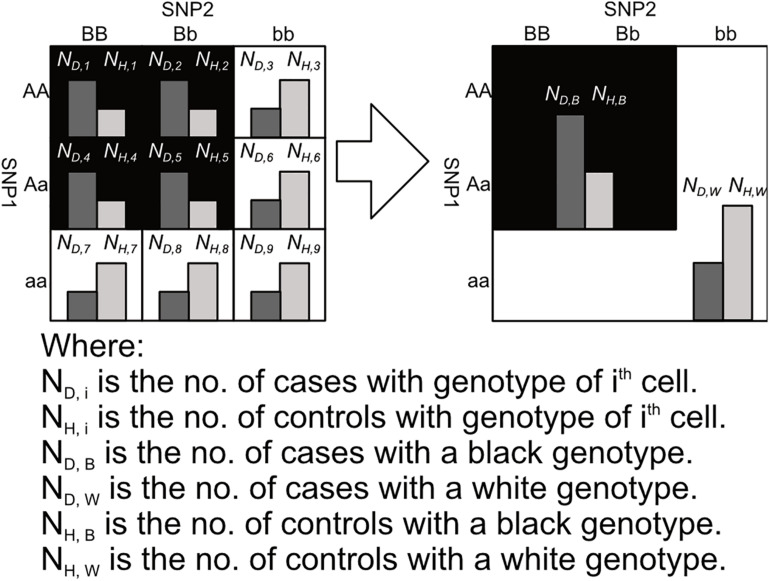
This figure shows the process of aggregating the number of cases and controls with black and white genotypes in a 3 × 3 table between SNP_1_ and SNP_2_, where the genotypes are colored according to the pattern 1 in Figure 2. Major alleles represented by upper-case letters (i.e., A, B) and minor alleles represented by lower-case letters (i.e., a, b) (Lee et al., 2018).

**FIGURE 4:**
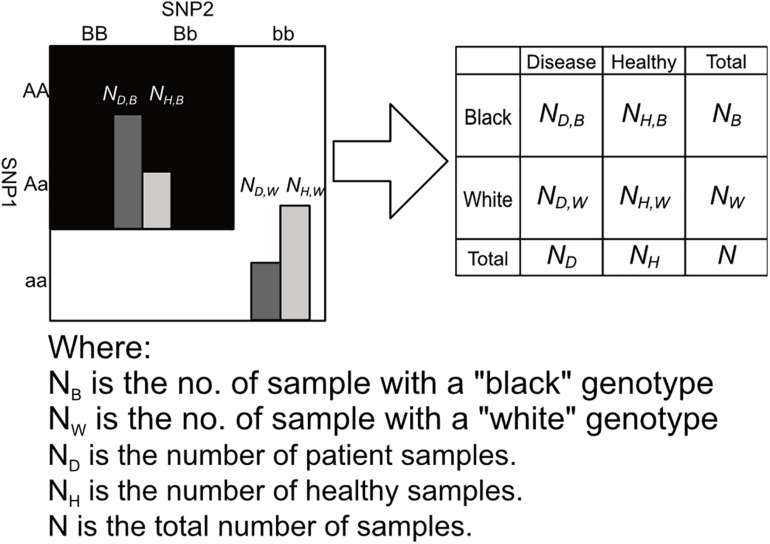
This figure shows the process of arranging the summed counts of cases and controls calculated in figure into a 2 × 2 contingency table. Major alleles represented by upper-case letters (i.e., A, B) and minor alleles represented by lower-case letters (i.e., a, b) (Lee et al., 2018).

After labeling the 3 × 3 genotype table of a 2nd order SNP combination, it can then be transformed into a 2 × 2 contingency table shown in Figures 3, 4. Among the black cells of the 3 × 3 genotype table at the left-hand side of Figure 3, the number of cases and controls are aggregated into the total number of cases *(N_*D,B*_)* and Controls *(N_*H,B*_)* respectively as shown in the table at the right-hand side of Figure 3. Similarly, the number of cases and controls of white genotypes are aggregated into *N*_*D,W*_ and *N_*H*,__*W*_* respectively. After calculating the aggregated number of cases and controls under different cell colors *(N_*D,B*_, N_*H,B*_, N_*D,W*_ and N_*H,W*_)*, a *2 × 2* contingency table can then be generated as shown in Figure 4.

After the corresponding 2 × 2 contingency tables of a 2nd order SNP combination *(SNP_*i*_*, *SNP_*j*_)* is calculated, statistical test like 1 d.f. chi-square statistical test can be performed to calculate the pairwise *p*-value of *SNP*_*i*_ and *SNP*_*j*_.

### Source of Real Datasets

We downloaded three schizophrenia GWAS datasets (phs000021:phg000013, phs000021:phg000014 and phs000167) from the database of Genotypes and Phenotypes (dbGaP). These three datasets were cleansed to remove low quality SNPs and samples with Plink (Purcell et al., 2007) following the common recommendations from NCBI (Anderson et al., 2010). The cleansing parameters are shown in Supplementary Tables S1, S2. The demographic information of the three datasets are shown in Supplementary Tables S4–S6.

After data pre-processing, there were 729,454 SNPs and 2,306 samples (cases: 1,051, controls: 1,214) in dataset phs000021:phg000013; 761,628 SNPs and 1,726 samples (cases: 829, controls: 874) in dataset phs000021:phg000014; 767,002 SNPs and 2689 samples (cases: 1,176, controls: 1,325) in dataset phs000167. The genotypes of every SNP in these three datasets were encoded as 0, 1, 2, 3 according to the encoding scheme shown in Supplementary Table S3.

### Exhaustive Search on Schizophrenia Datasets in Discovery Dataset and 2 Replication Datasets

To identify potential biologically plausible and statistically significant 2nd order SNP–SNP interactions, we performed search on these three cleansed datasets. As phg000013 got a slightly larger sample size and was restricted to Caucasian subjects, it was chosen as the discovery dataset and the other two were used for replication of findings obtained from phg000013. First, we performed our search on these three datasets after filtering out SNPs in chromosome X, Y and mitochondrial DNA. After SNP–SNP interactions were ranked by their pairwise *p*-value, high ranking interactions would be selected for further analysis.

After sorting SNP–SNP interactions found in the discovery datasets in terms of their *p*-value, only a handful of interactions found in datasets phs000021:phg000013 had a *p*-value better than 10^–11^ which could be used as a cut-off value for statistically significant and the top 10 pairs of interaction were listed in Supplementary Material. They were largely related to SLC35A5 and an unknown transcript LOC105375629. However, the interaction was not replicated in the other 2 datasets.

#### Enrichment of Interacting Genes Among Top 10,000 Interactions

We considered using the Bonferroni approach for correction of cut-off *p*-values was too conservative and important interactions would be missed. Then we looked at the extend of replication among top ranked interaction found in the discovery dataset. Five sets of top ranked interaction found in phg000013 were checked if their component genes were also found to have high ranking in the two replication datasets (Supplementary Tables S9, S10). From the 2 tables, it was clear that up to 30% of genes reported in the top 10,000 interaction list could be replicated. This percentage replication (labeled as % common in the Supplementary Tables) increased with increasing number of top ranked interaction selected. The results indicated that signal were in fact enriched in the top ranked interaction SNPs pairs though they were not significant by Bonferroni correction. The very extreme *p*-values in the Supplementary Tables represents the probability of null hypothesis that there was no signal enrichment. We tried an addition way of sample filtering to exclude data bias or confounding by limiting to using female only subjects and repeating the whole procedure. Indeed, the same signal enrichment results was found among the top 10000 interacting gene pairs. Therefore, in the subsequent replication analysis, we selected top 10000th SNP–SNP interactions in terms of *p*-value.

After that, we selected *n^*th*^ (n = 100, 500, 1000, 5000, 10000)* SNP–SNP interactions in terms of *p*-value from each dataset after each stage of our experiment and we predicted gene–gene interactions from these SNP–SNP interactions with CADD (Kircher et al., 2014). Then, we compared the gene-gene interactions predicted from the results of different datasets and analyzed the consistency among different datasets. Furthermore, we inferred and analyzed the biological function of these gene–gene interactions with GSEA (Mootha et al., 2003; Subramanian et al., 2005). Meanwhile, we constructed gene networks based on the gene–gene interactions which have a potential biological function based on our analysis with GSEA. Then, we performed follow-up network analysis on these gene networks. Genes which had a high degree within our network are selected for further analysis to identify potential novel schizophrenia associated genes.

#### Replication of SNP–SNP Interaction in Other 2 Datasets

Based on the top 10,000 SNP–SNP interaction pairs found discovery datasets, they were analyzed in the two replication datasets to see if they were also among the top 10,000 interaction. For those replicated interactions, odd ratios, *p*-values and 3 × 3 genotype data were shown. Bioinformatic methods were used to explore the potential biology of these replications including analysis of non-coding RNA binding sites, GO terms and gene set enrichment analysis.

According to existing literature, gene-gene interactions could be discovered through analyzing SNPs which are acting as conditional eQTLs (Jansen et al., 2017). After retrieving the genomic position of every SNP through referring the genome assembly GRCh37 published by Genome Reference Consortium (Schneider and Church, n.d.), the gene closest to every component SNP of every SNP-SNP interaction (if available) could then be found with CADD version 1.4 (Kircher et al., 2014). By making an assumption that if *SNP*_*i*_ and *SNP*_*j*_ were having a SNP–SNP interaction, *Gene*_*i*_ and *Gene*_*j*_ would have a corresponding gene-gene interaction (where *Gene*_*i*_ and *Gene*_*j*_ are the genes closest to *SNP*_*i*_ and *SNP*_*j*_ respectively), gene-gene interactions could then be predicted from the SNP–SNP interactions which we found.

#### Analysis of Protein Coding Transcript and Non-coding Transcripts

If there were non-coding genes closest to the SNP-SNP interaction pairs, LncRRIsearch (Fukunaga et al., 2019) was used to predict the lncRNA–RNA interactions of the corresponding gene–gene pairs. Threshold interaction energy was set to -12 kcal/mol here. To see whether the lncRNA–RNA interaction was specific, we used LncRRIsearch to investigate the interaction of the mRNA with 10 adjacent lncRNAs located both upstream and downstream of the interacting lncRNA.

## Results and Discussion

### Analysis on Top 10 SNP–SNP Interactions Found in Discovery Dataset

To reduce bias and confounding, we tried to analysis the discovery dataset in two different stages by using two sample filters. In our stage one experiment, we performed exhaustive search after filtering out SNPs in chromosome X, Y and mtDNA. Interactions were then ranked according to their *p*-value. Top 10 SNP–SNP interactions are listed in Supplementary Table S7. In the discovery dataset phs000021:phg000013, there were six SNPs rs7819913, rs1580508, rs16884273, rs35385383, rs16884251, and rs35648 reported to be associated to schizophrenia (Glessner et al., 2010; Bigdeli et al., 2016). On the other hand, there was a SNP rs34165590 being as an eQTL of a gene MMP16 in tibial nerve tissue according to GTEx Portal (Lonsdale et al., 2013). This gene was known to be associated to schizophrenia (Bitanihirwe et al., 2016). Meanwhile, there was a SNP rs8463 located in the 3-prime UTR region of gene RBM17 and this gene was known to be related to neurodegenerative diseases. There were two SNPs rs34729156 and rs1755286 which were located in the intron region of two schizophrenia-associated genes RYR2 and ADAMTSL1 respectively.

In our stage two experiment, we performed exhaustive search only using female samples and every SNP in autosomal chromosomes and chromosome X. Interactions were then ranked according to their *p*-value. Top 10 SNP–SNP interactions were selected from each dataset for further analysis and are listed in Supplementary Table S8. In the discovery dataset phs000021:phg000013, there were two SNPs rs10926030 and rs6050455 already reported in GTEx portal (Lonsdale et al., 2013) as expression quantitative trait loci (eQTls) of three genes FMN2, NOP56 and TMC2 in tibial nerve tissue where SNP rs10926030 acted as an eQTL of gene FMN2 and SNP rs6050455 acted an eQTL of genes NOP56 and TMC2. These three genes were known to be associated to schizophrenia and other neurodegenerative disorders (Mulligan et al., 2010; Kobayashi et al., 2011; Pamphlett et al., 2011; Van Scheltinga et al., 2013; Zhang et al., 2018). On the other hand, there were three SNPs rs12777747, rs41453047, and rs11755127 which were located in the intron region of the three literature-reported schizophrenia-associated genes TACC2, SEMA3A, and RPS6KA2 respectively. There were two SNPs rs8061891 and rs8057600 which were both located in the intron region of a schizophrenia-associated gene RBFOX1. We believed that these SNPs might have the potential to be associated to schizophrenia.

However, as it was more evidence in the female only analysis, genes or SNPs that were already significant predisposition gene by itself were mostly ranked among the top 10. For example, rs1277747 of TACC2 got a *p*-value of 2.03 × 10^–7^ on single SNP association analysis. In another word, many SNPs were also significant by itself and formed one of the interacting partner. In fact, 7 out of top 10 interacting pair was formed by exactly the same SNP, rs1277747. We believed that such GWAS significant SNP would dominate the interaction analysis and masked the true interacting pairs as they ranked lower in the list.

### Predicting Gene–Gene Interactions After Annotating SNPs With CADD

We selected the top n^*th*^ (*n* = 100, 500, 1000, 5000, 10000) SNP–SNP interactions in terms of *p*-value from each stage of our experiment independently and the corresponding gene-gene interactions of these SNP–SNP interactions could then be derived. First, we counted the number of gene-gene interactions predicted from each dataset and the number of individual component genes involved in those interactions. Then, we compared the gene–gene interactions predicted from each dataset against every other dataset and the number of common gene-gene interactions and common component genes between every pair of datasets were counted accordingly. The *p*-value on the number of occurrences of gene-gene interactions given the null hypothesis of no enrichment of signal were also calculated. Furthermore, the proportion of common component genes and the proportion of common gene-gene interactions in each dataset were calculated. These results are shown in Supplementary Tables S9, S10. Through observing the *p*-value on the number of common component genes, we believed that the occurrence of common component gene among datasets could not be simply explained as random cooccurrence by chance and genuine interaction signals must be enriched in the top ranked list. Furthermore, we observed that the proportion of common component genes under different pairs of datasets is roughly increasing linearly with the number of top ranked n^*th*^ interactions selected until *n* = 1000. When *n* = 5000, the increase trends in the proportion of common component genes under different pairs of datasets might start to reach the plateau. Therefore, we believed that top 10000^*th*^ of SNP–SNP interactions should already cover most of disease-associated SNP–SNP interactions in all three datasets. The top 10,000 gene–gene interactions found in the discovery dataset were visualized as circos diagrams as shown in Supplementary Figure S1. It was obvious that most gene–gene interactions were inter-chromosomal interactions.

### Analysis on Predicted Gene–Gene Interactions With GSEA

Every component gene *G* in every predicted gene-gene interaction was annotated through the Molecular Signatures Database of Gene Set Enrichment Analysis (GSEA) such that every functional gene set containing *G* could be found. In our analysis, if both component genes of a gene-gene interaction *I* belonged to a common functional gene set *F*, then we believe that interaction *I* was associated to the biological function represented by functional gene set *F*. Among the gene-gene interactions predicted from top n^*th*^ (*n* = 100, 500, 1000, 5000, 10000) SNP–SNP interactions, the number of predicted gene–gene interactions associated to each GSEA functional gene set was counted and shown in Supplementary Table S11. There were several predicted gene–gene interactions found to be associated to the following three functional gene sets namely GO_NEURON_PART, GO_NEURON_PROJECTION, and GO_SYNAPSE in stage one experiment of the discovery dataset. These gene–gene interactions were believed to be associated to the nervous system functionality and associated to schizophrenia.

Gene–gene interactions, which were considered to be associated to at least one biological functions in our analysis, were selected for forming gene networks. A gene–gene interaction could be selected for forming gene network only if both of its component genes were commonly predicted across three datasets. A gene network was then constructed for based on our results in the stage one experiment. Top 10 genes in terms of degree were separately selected from these two networks where the degree of a gene was the number of other neighboring genes directly interacting with it. These genes were listed in Supplementary Table S12. For each gene *G* in this table, direct interactions involving *G* were validated by the following three external biological interaction databases StringDB (Jensen et al., 2009), BioGrid (Stark et al., 2006) and RNAInter (Lin et al., 2020). A direct interaction was considered to be validated if there was at least one direct or indirect interactions between its two component genes in the external biological interaction databases. For each gene *G* and external biological database *D*, the number of validated direct interactions involving *G* and the mean and variance of the number of hidden interactors of these validated interactions under *D* were calculated.

Among the top 10 genes in terms of its degree selected from the network constructed based on the results of our stage one experiment, there were seven genes (CTNND2, ASTN2, DAB1, CAMK1D, PTPRD, RUNX1, ROBO2) which were reported to be associated to schizophrenia in existing literature as shown in Supplementary Table S12. This indicated schizophrenia-associated genes could be discovered from this network. Gene WDR27, VIT and CLSTN2 had not been previously reported to be associated with schizophrenia. However, these three genes were reported to be associated to insomnia (Hammerschlag et al., 2017), human brain asymmetry (Tadayon et al., 2016) and memory performance (Preuschhof et al., 2010) respectively. Therefore, they might all be associated to schizophrenia. This indicated GWAS discovered schizophrenia predisposition genes could also be discovered by our interaction analysis, however, this was not the primary aim of our analysis.

### List of Replicated SNP–SNP Interactions in the Three Datasets and Gene–lncRNA Interactions

Using phg000013 as discovery dataset and the other 2 as replication datasets, the top 10,000 interaction pairs were mined to see if they were replicated in other datasets. Only replication by the exactly identical pattern out the 8 interactions were counted. After filtering out interactions with at least one component SNP which could not be mapped to a gene according to CADD, 14 interactions were replicated in both replication datasets in the exactly the same way as in the discovery dataset. These interactions were shown in Table 1.

**TABLE 1:** This table shows the *p*-value and odds ratio of the 14 SNP–SNP interactions found in discovery dataset phs000021:phg000013 which are all replicable in both replication datasets phs000021:phg000014 and phs000167.

SNP1	SNP2	Pattern	Discovery		Replication			
			phs000021: phg000013		phs000021: phg000014		phs000167	
			*P*-Value	Odds Ratio	*P*-Value	Odds Ratio	*P*-Value	Odds Ratio
rs2638037	rs7819913	4	3.54 × 10^–10^	3.92	0.0480	1.22	0.00729	1.56
rs2638037	rs1580508	4	3.54 × 10^–10^	3.92	0.0480	1.22	0.00729	1.56
rs1873571	rs35385383	4	3.73 × 10^–10^	3.92	0.0476	1.22	0.00908	1.54
rs2638037	rs35385383	4	3.86 × 10^–10^	3.91	0.0448	1.22	0.00705	1.56
rs7735699	rs2755145	5	1.59 × 10^–9^	1.88	0.0479	1.23	0.00267	1.33
rs16867416	rs7026201	7	4.58 × 10^–9^	2.69	0.0429	1.51	0.0458	1.35
rs7735699	rs2755152	5	6.91 × 10^–9^	1.84	0.0412	1.25	0.00545	1.31
rs4704591	rs2755145	5	8.09 × 10^–9^	1.83	0.0404	1.23	0.00236	1.34
rs17746902	rs9635370	4	8.77 × 10^–9^	3.52	0.0316	1.23	0.0346	1.42
rs668805	rs11591783	4	1.30 × 10^–8^	1.78	0.00400	1.63	0.0229	1.25
rs3856662	rs2550266	4	1.38 × 10^–8^	2.01	0.0456	1.45	0.0141	1.32
rs585870	rs11591783	4	1.38 × 10^–8^	1.78	0.00301	1.56	0.0403	1.22
rs9556688	rs4822752	1	1.39 × 10^–8^	1.66	0.0397	1.28	0.0268	1.21
rs16867416	rs17680408	1	1.50 × 10^–8^	1.89	0.0343	1.42	0.0400	1.24

While we and most researchers are expecting to find protein interacting partners and investigate our discoveries with protein-protein interaction databases such as StringDB (Jensen et al., 2009), PINBA (Yu et al., 2014), and DMS (Jia et al., 2011) in this kind of analysis, 11 out of 14 replicated interactions involved gene transcript with no protein product, or non-coding RNA. 3 of them are recognized long non-coding RNA (lncRNA), including LINC01934 (AC104820.2), MUC20-OT1 (LINC00969), and LINC00456 (Table 2).

**TABLE 2:** This table summarizes our analysis on the gene–gene interactions predicted from the 14 SNP–SNP replicable interactions.

Gene1 (SNP1)	Gene2 (SNP2)	Gene2 is lncRNA	Alias for Gene2	lncRNA (Gene2) | RNA (Gene1) interaction	Specific lncRNA | RNA interaction	Both Gene1 and Gene2 expressed in Brain
FHIT (rs3856662)	LINC00969 (rs2550266)	Yes	MUC20-OT1	Yes	Yes	Yes
ATG3 (rs2638037)	RP11-586K2.1 (rs7819913)	Yes	AC090578.1	Yes	No	Yes
ATG3 (rs2638037)	RP11-586K2.1 (rs1580508)	Yes	AC090578.1	Yes	No	Yes
ATG3 (rs1873571)	RP11-586K2.1 (rs35385383)	Yes	AC090578.1	Yes	No	Yes
ATG3 (rs2638037)	RP11-586K2.1 (rs35385383)	Yes	AC090578.1	Yes	No	Yes
VPS41 (rs17680408)	AC104820.2 (rs16867416)	Yes	LINC01934	Yes	No	No
C9orf171 (rs7026201)	AC104820.2 (rs16867416)	Yes	LINC01934	No	—	—
ABTB2 (rs2755145)	CTC-431G16.2 (rs7735699)	Yes	AC008496.2	No	—	—
ABTB2 (rs2755152)	CTC-431G16.2 (rs7735699)	Yes	AC008496.2	No	—	—
ABTB2 (rs2755145)	CTC-431G16.2 (rs4704591)	Yes	AC008496.2	No	—	—
CRYBB1 (rs4822752)	LINC00456 (rs9556688)	Yes	LINC00456	No	—	—
PEX14 (rs668805)	TCERG1L (rs11591783)	No	—	—	—	—
PEX14 (rs585870)	TCERG1L (rs11591783)	No	—	—	—	—
BIN3 (rs17746902)	PAQR5 (rs9635370)	No	—	—	—	—

*The alias of each lncRNA gene is shown in this table. Furthermore, interactions which involved gene transcript with no protein product, or non-coding RNA and interactions where both genes were found to be expressed in brain are marked accordingly.*

**FIGURE 5:**
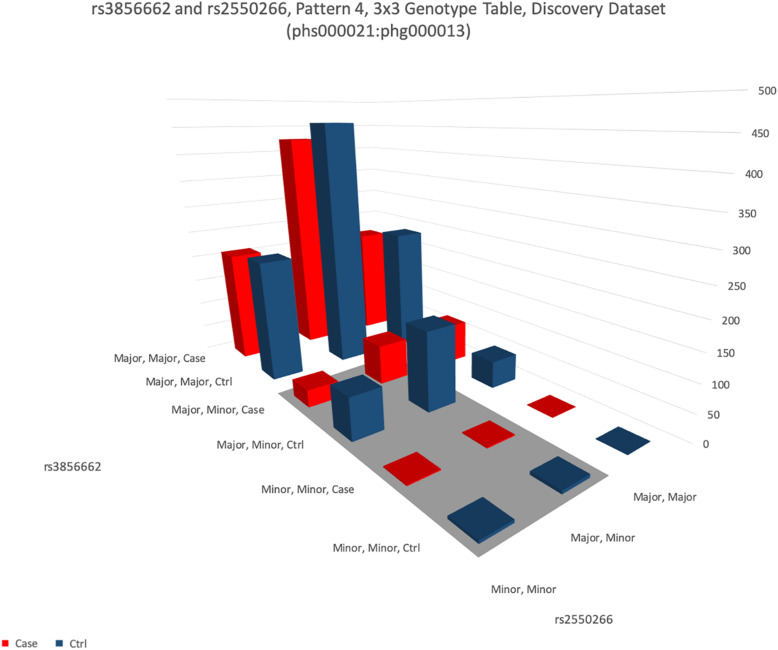
This figure shows the distribution of genotypes of two SNPs rs3856662 (FHIT gene) and rs2550266 (LINC00969). The height of the bar represents the number of samples of each of the nine combinations of the genotypes in these two SNPs. For each genotype combination, the bar in blue color represents the number of healthy samples (controls) and the bar in red color represents the number of patients (cases). It is apparent that the four blue (control) bars in the shaded area (dominant-dominant interaction between minor alleles of both SNPs) are over-represented than the four red (case) bars.

**FIGURE 6:**
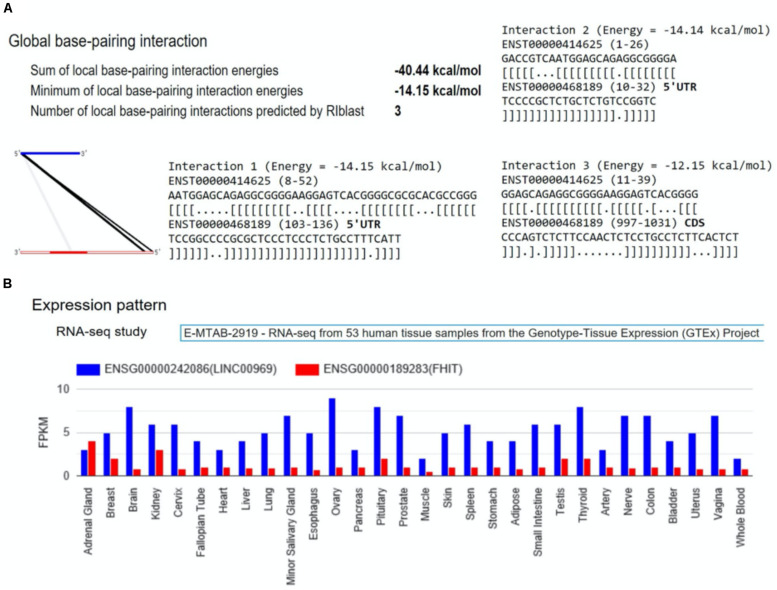
The output of LncRRIsearch (Fukunaga et al., 2019) for the interaction of the transcripts of LINC00969 and FHIT. **(A)** The graphical and text view of the global base -pairing interactions of LINC00969 and FHIT predicted by RIblast (Fukunaga and Hamada, 2017); **(B)** The expression pattern of LINC00969 and FHIT in RNA-seq data of GTEx consortium (E-MTAB-2919) (Lonsdale et al., 2013). LINC00969 and FHIT were represented by blue color and red color respectively.

Therefore, we detailly investigated if there was evidence of binding sites between each of these coding transcripts and lncRNA transcripts using LncRRIsearch (Fukunaga et al., 2019). Six SNP–SNP interactions were found to have lncRNA–RNA interactions in their associated genes: rs16867416 (LINC01934, alias AC104820.2) and rs17680408 (VPS41), rs7819913 (AC090578.1, alias RP11-586K2.1) and rs2638037 (ATG3), rs1580508 (AC090578.1, alias RP11-586K2.1) and rs2638037 (ATG3), rs35385383 (AC090578.1, alias RP11-586K2.1) and rs1873571 (ATG3), rs35385383 (AC090578.1, alias RP11-586K2.1) and rs2638037 (ATG3), rs3856662 (FHIT) and rs2550266 (MUC20-OT1, alias LINC00969) (Figure 5). The sum of local base-pairing interaction energies of lncRNA-RNA interactions in LINC01934 (AC104820.2)-VPS41, AC090578.1 (RP11-586K2.1)-ATG3 and LINC00969 (MUC20-OT1)- FHIT gene pairs are −38.97 kcal/mol (3 interactions), −12.20 kcal/mol and −40.44 kcal/mol (3 interactions) respectively. As it is based on the principle of alignment entropy, it is difficult to assess how specific are these *in silico* identified binding sites. We screened the adjacent 10 lncRNAs located both upstream and downstream in the chromosomal region of the specific interacting lncRNA to see if these adjacent lncRNAs might interact with the protein-coding gene and its mRNA transcript. Among the three lncRNA–RNA interactions, only LINC00969-FHIT interaction is specific, with the mRNA of FHIT showing no interaction or weaker interaction with the adjacent lncRNAs of LINC00969 (Table 3 and Figure 6A). Furthermore, the tissue expression profiles of both the coding transcript and lncRNA were examined. The interaction is only plausible if both are expressed in the same tissue and more specific, brain or neuronal tissues. According to the LncRRIsearch result (Figure 6B), both LINC00969 and FHIT are expressed in multiple tissues at the same time, especially brain tissue, based on the RNA-seq data of GTEx consortium (E-MTAB-2919) (Lonsdale et al., 2013). The above analysis is summarized and showed in Table 2.

**TABLE 3:** Interaction of transcript of FIHT (ENST00000468189) and 10 lncRNA near the upstream or downstream of the LINC00969 (MUC20-OT1) based on LncRRIsearch (Fukunaga et al., 2019).

Nearby lncRNA	Ensemble ID	Chromosome	Start position	Hgnc symbol	Sum of interaction energies	Expressed in brain#
Downsteam_10	ENSG00000287073	3	194708010		ID is not found*	NA
Downsteam_9	ENSG00000237222	3	194708093	LINC01968	−12.42 kcal/mol	No
Downsteam_8	ENSG00000230401	3	194765238	LINC01972	No interaction	NA
Downsteam_7	ENSG00000238031	3	194827890		No interaction	NA
Downsteam_6	ENSG00000233303	3	195094588	XXYLT1-AS1	No interaction	NA
Downsteam_5	ENSG00000230266	3	195147871	XXYLT1-AS2	No interaction	NA
Downsteam_4	ENSG00000287005	3	195260632		ID is not found	NA
Downsteam_3	ENSG00000229325	3	195280723	ACAP2-IT1	No interaction	NA
Downsteam_2	ENSG00000223711	3	195544048		No interaction	NA
Downsteam_1	ENSG00000229178	3	195655565		No interaction	NA
Selected_LncRNA	ENSG00000242086	3	195658062	MUC20-OT1	−40.44 kcal/mol (3 interactions)	Yes
Upstream_1	ENSG00000223783	3	195836193	LINC01983	No interaction	NA
Upstream_2	ENSG00000286004	3	195900986		No interaction	NA
Upstream_3	ENSG00000224614	3	195908076	TNK2-AS1	No interaction	NA
Upstream_4	ENSG00000286168	3	195996262		ID is not found	NA
Upstream_5	ENSG00000224652	3	196142525	LINC00885	−38.26 kcal/mol (3 interactions)	NA
Upstream_6	ENSG00000228028	3	196250542		No interaction	NA
Upstream_7	ENSG00000235897	3	196318330	TM4SF19-AS1	−12.12 kcal/mol	NA
Upstream_8	ENSG00000225822	3	196431385	UBXN7-AS1	-12.91 kcal/mol	Yes
Upstream_9	ENSG00000273013	3	196474801		No interaction	NA
Upstream_10	ENSG00000286661	3	196598549		ID is not found	NA

ID is not found* means the lncRNA was not included in the database of LncRRIsearch. Expressed in brain#; The expression level of the lncRNA in brain samples was investigated based on the RNA-seq data of GTEx consortium (E-MTAB-2919) (Lonsdale et al., 2013) if the lncRNA has interaction with FIHT.

A notable results was interaction between rs3856662 (FHIT gene) and rs2550266 (LINC00969). The 3 × 3 genotype table of this interaction is visualized in Figure 5, while the 3 × 3 genotype table of the other 13 replicable interaction are visualized as Supplementary Figures S2–S14. Statistical analysis of the interaction indicated that minor alleles of both gene interacted to reduce the risk of schizophrenia in a dominant way, such that carriers of both minor alleles of both genes had a 50% reduction in risk for schizophrenia (odds ratio = 0.50, confidence interval: 0.39–0.64). Combined *p*-values from 3 datasets were 3.15 × 10^–9^. Binding sites analysis were confirmed by both lncRNA software we used. It was possible for LINC00969 to bind to transcribed region of FHIT with a good predicted value of entropy.

Interaction of this profile might be genuine and representing how real interaction would look like. Genuine interaction profile should have these characteristics: (1) neither SNPs is significant by itself, or both interacting SNPs are not GWAS hits, (2) interaction should have a stronger effect size than single SNP GWAS hits, which usually have odds ratios in the range of 1.1–1.2 and (3) replication of interaction is evident. The example of rs3856662 (FHIT gene) and rs2550266 (LINC00969) indeed fulfills these expectations. Firstly, neither of them was significant by single SNP, and single SNP association *p*-values were not significant at only 5 × 10^–3^ and 1 × 10^–1^. Secondly, the interaction protective effect was 0.5 (effect size is equivalent to odds ratio of 2). So such genetic risk profile is more useful in clinical setting. Finally, the interaction was observed in all 3 datasets. It is of note that replication was seen across ethnic groups (as phg000014 was an African dataset). The reduction of risk could be found even though the allelic frequencies of rs2550266 were very different between Africans and Caucasians. The pan-ethnic variation of allele frequency can be found in dbSNP^1^. Other potential interacting SNP pairs that could be replicated in one dataset but not both were shown in Supplementary Table 13.

## Conclusion

In conclusion, after exhaustively search for every 2nd order SNP-SNP interaction from the discovery schizophrenia dataset phs000021:phg000013 with eight different biological plausible SNP–SNP interaction patterns, we first reported here that 9 out the top 10 SNP-SNP interactions in terms of *p*-value might represent interactions between protein coding genes and long non-coding RNA (lncRNA) genes. These results indicate the importance of interactions of other bio-molecules (like lncRNA–Protein, lncRNA–RNA, etc.) in addition to that of the traditional protein–protein interactions. Then, we showed there was a high proportion of concurrent genes among the gene–gene interactions predicted from the top 10 SNP–SNP interaction in terms of *p*-value. Therefore, these interactions were replicable under gene-level analysis and there was a strong enrichment of signals among these interactions. Finally, 9 SNP–SNP interactions were successfully replicated in both replication datasets. We discovered that one of these SNP–SNP interactions can be interpreted as the interaction between FHIT (protein coding) and LINC00969 (lncRNA). Both FHIT and LINC00969 were reported to have expression in brain and might be a new discovery.

## Data Availability Statement

Publicly available datasets were analyzed in this study. This data can be found here: http://www.ncbi.nlm.nih.gov/gap.

## Author Contributions

K-YL conducted the project, developed the algorithms used in this study, and ran the experiments under the supervision of M-HW and K-SL. K-YL and DH interpreted the biological meaning of the experimental results under the guidance of NT and HS. K-YL, SM, and DH wrote the main manuscript text. All the authors discussed the results and reviewed the manuscript thoroughly.

## Conflict of Interest

NT and K-SL are directors and hold shares of the company Cytomics Ltd. Cytomics Ltd holds a licence to use the patent related to single cell type specific gene expression analysis. The remaining authors declare that the research was conducted in the absence of any commercial or financial relationships that could be construed as a potential conflict of interest.

## Footnotes

1 https://www.ncbi.nlm.nih.gov/snp/rs2550266#frequency_tab
