# Supplementary material for: Genome-Wide Search for SNP Interactions in GWAS Data: Algorithm, Feasibility, Replication Using Schizophrenia Datasets
Source: Front Genet. 2020 Aug 28;11:1003. doi: 10.3389/fgene.2020.01003 (PMC7505102; doi:10.3389/fgene.2020.01003)
Supplement: Supplementary file 1 [file Data_Sheet_1.zip › Data Sheet.PDF]

Supplementary Material

	phs000021		phs000167
	phg000013	phg000014	
Missing rate	>0.01		>0.1
Minor allele frequency (MAF)	<0.01		
Hardy-–Weinberg equilibrium (HWE)	*p*-value<0.00001		
Missing rate deviation between cases and controls	*p*-value<0.00001		

Supplementary Table 1. This table shows the data cleansing parameter for filtering out low-quality SNPs. SNPs satisfying any one of the following conditions listed in this table are excluded.

	phs000021		phs000167
	phg000013	phg000014	
Missing rate	>0.01	>0.01	>0.03
Gender	Inconsistency between reported gender and gender predicted with X chromosome		
Heterozygosity rate	<0.315 or >0.325	<0.29 or >0.32	<0.25 or >0.30

Supplementary Table 2. This table shows the data cleansing parameter for filtering out low-quality samples. Samples satisfying any one of the following conditions listed in this table are excluded.

Original Genotype	Encode Value
Missing Data	0
Major Allele, Major Allele	1
Major Allele, Minor Allele	2
Minor Allele, Minor Allele	3

Supplementary Table 3. This table shows the genotype encoding scheme of every SNP for a GWAS dataset.

	Case		Control	
Sex	Count	%	Count	%
Male	850	69.959	668	46.325
Female	365	30.041	774	53.675
Age	Mean	S.D.	Mean	S.D.
At interview/sampling	43.687	11.310	51.188	16.978
At onset	20.972	6.729	-	-
Diagnosis	Count	%	Count	%
Schizophrenia	1071	88.148	-	-
Schizoaffective depressed disorder	58	4.774	-	-
Schizoaffective bipolar disorder	86	7.078	-	-

Supplementary Table 4. This table shows the demographic information of dataset phs000021:phg000013 which only contains samples with European ancestry

	Case		Control	
Sex	Count	%	Count	%
Male	594	62.329	370	37.794
Female	359	37.671	609	62.206
Age	Mean	S.D.	Mean	S.D.
At interview/sampling	43.268	10.220	45.443	13.223
At onset	21.021	6.857	-	-
Diagnosis	Count	%	Count	%
Schizophrenia	867	90.976	-	-
Schizoaffective depressed disorder	43	4.512	-	-
Schizoaffective bipolar disorder	43	4.512	-	-

Supplementary Table 5. This table shows the demographic information of dataset phs000021:phg000014 which only contains samples of African American

	Case		Control	
Sex	Count	%	Count	%
Male	876	69.304	700	50.542
Female	388	30.696	685	49.458
Self-ident-ancestry	Count	%	Count	%
African American	85	6.725	21	1.516
European Ancestry	1179	93.275	1364	98.484
Age	Mean	S.D.	Mean	S.D.
At interview	42.842	11.905	49.823	15.739
At onset	21.715	7.281	-	-
Diagnosis	Count	%	Count	%
Schizophrenia	1126	89.082	-	-
Schizoaffective depressed disorder	58	4.589	-	-
Schizoaffective bipolar disorder	80	6.329	-	-

Supplementary Table 6. This table shows the demographic information of dataset phs000167

No.	SNP1	SNP1 Nearby Gene	SNP2	SNP2 Nearby Gene	Pattern	SNP1 *p*-value	SNP2 *p*-value	2nd Order *p*-value
1	rs766968	SLC35A5	rs34165590	LOC-105375629	4	3.05×10^{-4}	1.77×10^{-2}	1.74×10^{-11}
2	rs766968	SLC35A5	rs7819913	LOC-105375629	4	3.05×10^{-4}	3.49×10^{-2}	2.35×10^{-11}
3	rs766968	SLC35A5	rs1580508	LOC-105375629	4	3.05×10^{-4}	3.16×10^{-2}	2.35×10^{-11}
4	rs766968	SLC35A5	rs16884273	LOC-105375629	4	3.05×10^{-4}	3.61×10^{-2}	2.42×10^{-11}
5	rs766968	SLC35A5	rs35385383	LOC-105375629	4	3.05×10^{-4}	4.24×10^{-2}	2.59×10^{-11}
6	rs8463	RBM17[1,2]	rs35648	LOC-105375629	5	2.29×10^{-4}	2.55×10^{-5}	3.01×10^{-11}
7	rs766968	SLC35A5	rs16884251	LOC-105375629	4	3.05×10^{-4}	4.33×10^{-2}	3.91×10^{-11}
8	rs34729156	RYR2[3,4]	rs12572597	SLIT1	4	2.49×10^{-3}	2.12×10^{-3}	5.13×10^{-11}
9	rs1515497	TP63	rs34234	PDZPH1P	5	6.85×10^{-3}	1.33×10^{-3}	7.39×10^{-11}
10	rs4870327	LOC-100421330	rs1755286	ADAMTSL1[5]	6	5.73×10^{-2}	2.51×10^{-3}	7.97×10^{-11}

Supplementary Table 7. This table shows the top 10 2nd order SNP-SNP interactions in terms of pairwise *p*-value from our discovery dataset phs000021:phg000013 in our stage one experiment where SNPs in chromosome X, Y and MT are excluded. Genes associated to schizophrenia or other mental disorders are annotated with footnotes.

[1] Lim et al., "Opposing Effects of Polyglutamine Expansion on Native Protein Complexes Contribute to SCA1."
[2] Tan et al., "Extensive Cryptic Splicing upon Loss of RBM17 and TDP43 in Neurodegeneration Models."
[3] Costain et al., "Pathogenic Rare Copy Number Variants in Community-Based Schizophrenia Suggest a Potential Role for Clinical Microarrays."
[4] Kirov et al., "De Novo CNV Analysis Implicates Specific Abnormalities of Postsynaptic Signalling Complexes in the Pathogenesis of Schizophrenia."
[5] Narayan et al., "Molecular Profiles of Schizophrenia in the CNS at Different Stages of Illness."

No.	SNP1	SNP1 Nearby Gene	SNP2	SNP2 Nearby Gene	Pattern	SNP1 *p*-value	SNP2 *p*-value	2nd Order *p*-value
1	rs12777747	TACC2[1,2]	rs5755403	ISX-AS1	1	2.03×10^{-7}	6.78×10^{-5}	1.05×10^{-12}
2	rs41453047	SEMA3A[3,4]	rs12777747	TACC2	1	1.30×10^{-4}	2.03×10^{-7}	2.55×10^{-12}
3	rs10926030	LOC-105373224	rs12777747	TACC2	1	9.75×10^{-5}	2.03×10^{-7}	2.56×10^{-12}
4	rs7897660	LOC-105376387	rs12777747	TACC2	1	3.84×10^{-5}	2.03×10^{-7}	2.73×10^{-12}
5	rs11692103	FTH1P6	rs1363458	RN7SKP122	8	2.91×10^{-2}	7.66×10^{-3}	2.82×10^{-12}
6	rs12777747	TACC2[1,2]	rs6050455	TMC2	1	2.03×10^{-7}	1.05×10^{-4}	2.94×10^{-12}
7	rs10954100	LOC-105375487	rs6471368	LOC-107986956	6	1.30×10^{-1}	4.14×10^{-5}	4.70×10^{-12}
8	rs12777747	TACC2[1,2]	rs8061891	RBFOX1[5]	1	2.03×10^{-7}	3.09×10^{-4}	4.78×10^{-12}
9	rs12777747	TACC2[1,2]	rs8057600	RBFOX1[5]	1	2.03×10^{-7}	1.21×10^{-4}	5.02×10^{-12}
10	rs6712833	MIR3681HG	rs11755127	RPS6KA2[6]	6	1.85×10^{-1}	3.16×10^{-4}	5.13×10^{-12}

Supplementary Table 8. This table shows the top 10 2nd order SNP-SNP interactions in terms of *p*-value from our discovery dataset phs000021:phg000013 in our stage two experiment where only female samples are included. Genes associated to schizophrenia or other mental disorders are annotated with footnotes.

[1] Qin et al., "Meta-Analysis of Sex Differences in Gene Expression in Schizophrenia."
[2] Mistry, Gillis, and Pavlidis, "Genome-Wide Expression Profiling of Schizophrenia Using a Large Combined Cohort."
[3] Eastwood et al., "The Axonal Chemorepellant Semaphorin 3A Is Increased in the Cerebellum in Schizophrenia and May Contribute to Its Synaptic Pathology."
[4] Gilabert-Juan et al., "Semaphorin and Plexin Gene Expression Is Altered in the Prefrontal Cortex of Schizophrenia Patients with and without Auditory Hallucinations."
[5] Georgieva et al., "De Novo CNVs in Bipolar Affective Disorder and Schizophrenia."
[6] Meda et al., "Multivariate Analysis Reveals Genetic Associations of the Resting Default Mode Network in Psychotic Bipolar Disorder and Schizophrenia."

		Top N^{th} SNP-SNP interactions selected				
		100	500	1000	5000	10000
Stage 1 (No Chr X/Y/MT)	phs000021:phg000013	60	209	388	1260	2067
	phs000021:phg000014	63	253	444	1654	2789
	common	1	8	27	242	584
	% common in phg000013	1.67%	3.83%	6.96%	19.21%	28.25%
	% common in phg000014	1.59%	3.16%	6.08%	14.63%	20.94%
	p-value	6.67×10^{-2}	6.52×10^{-8}	3.00×10^{-17}	4.89×10^{-122}	8.06×10^{-278}
Stage 2 (Female Only)	phs000021:phg000013	51	204	381	1583	2793
	phs000021:phg000014	66	260	469	1814	3173
	common	1	10	28	343	849
	% common in phg000013	1.96%	4.90%	7.35%	21.67%	30.40%
	% common in phg000014	1.52%	3.85%	5.97%	18.91%	26.76%
	p-value	5.67×10^{-2}	3.69×10^{-8}	2.10×10^{-18}	3.81×10^{-187}	0

Supplementary Table 9. This table shows the total number of independent component genes participating in the gene-gene interactions predicted from top N (N =100, 500, 1000, 5000, 10000) SNP-SNP interactions in terms of *p*-value found in our discovery dataset phs000021:phg000013 and our replication dataset phs000021:phg000014 from our two stage experiment. The number of common component genes across both datasets are listed in this table. For each dataset, the proportion of component genes which are commonly found in both datasets is also listed in this table.

		Top N^{th} SNP-SNP interactions selected				
		100	500	1000	5000	10000
Stage 1 (No Chr X/Y/MT)	phs000021:phg000013	60	209	388	1260	2067
	phs000167	36	145	304	1140	2014
	common	2	5	19	190	452
	% common in phg000013	3.33%	2.39%	4.90%	15.08%	21.87%
	% common in phs000167	5.56%	3.44%	6.25%	16.67%	22.44%
	p-value	7.24×10^{-4}	2.48×10^{-4}	9.92×10^{-13}	4.32×10^{-105}	2.63×10^{-223}
Stage 2 (Female Only)	phs000021:phg000013	51	204	381	1583	2793
	phs000167	37	168	331	1232	2123
	common	0	4	22	244	583
	% common in phg000013	0%	1.96%	5.77%	15.41%	20.87%
	% common in phs000167	0%	2.38%	6.65%	19.81%	27.46%
	p-value	-	3.09×10^{-3}	1.09×10^{-15}	1.28×10^{-135}	3.35×10^{-281}

Supplementary Table 10. This table shows the total number of independent component genes participating in the gene-gene interactions predicted from top N (N =100, 500, 1000, 5000, 10000) SNP-SNP interactions in terms of *p*-value found in our discovery dataset phs000021:phg000013 and our replication dataset phs000167 from our two stage experiment. The number of common component genes across both datasets are listed in this table. For each dataset, the proportion of component genes which are commonly found in both datasets is also listed in this table

	Top n^{th} SNP-SNP interactions selected				
	100	500	1000	5000	10000
AACTTT_UNKNOWN	0	1	2	14	28
CAGGTG_E12_Q6	0	0	2	8	19
CTTTGT_LEF1_Q2	0	1	1	8	14
GO_CELL_DEVELOPMENT	1	3	3	5	10
GO_CELL_PROJECTION	0	1	1	9	19
GO_NEURON_PART	0	0	0	5	14
GO_NEURON_PROJECTION	0	0	0	3	9
GO_SYNAPSE	0	1	1	2	8
GRYDER_PAX3FOXO1_ENHANCERS_IN_TADS	1	1	4	6	11
TTGTTT_FOXO4_01	1	2	2	9	16

Supplementary Table 11. This table shows the number of predicted gene-gene interactions where both of their component gene belonging to each particular GESA functional gene set. These gene-gene interactions are predicted from the top N (N =100, 500, 1000, 5000, 10000) SNP-SNP interactions in terms of *p*-value found in our discovery dataset phs000021:phg000013 under our stage one experiment where SNPs in chromosome X, Y and MT are excluded.

	Gene	Degree	StringDB			BioGrid			RNAInter		
			Valid	Number of Intermediate interactors		Valid	Number of Intermediate Interactors		Valid	Number of Intermediate interactors	
				Mean	Variance		Mean	Variance		Mean	Variance
1	WDR27	12	2	2.50	4.50	12	1.75	0.57	11	1.00	0.00
2	CTNND2[1]	8	5	1.40	0.80	8	2.00	0.29	8	0.75	0.21
3	ASTN2[1]	6	0	-	-	6	1.83	0.17	6	0.83	0.17
4	DAB1[2]	3	1	2.00	-	3	1.00	0.00	2	0.50	0.50
5	VIT	3	0	-	-	3	2.67	0.33	3	1.00	0.00
6	CAMK1D[3]	3	1	2.00	-	3	1.33	0.33	2	0.00	0.00
7	PTPRD[4]	3	0	-	-	3	2.00	0.00	3	1.00	0.00
8	CLSTN2	2	0	-	-	2	2.50	0.50	2	1.00	0.00
9	RUNX1[5]	2	0	-	-	2	2.00	0.00	2	0.50	0.50
10	ROBO2[6]	2	1	3.00	-	1	2.00	-	2	1.00	0.00

Supplementary Table 12. Among the common genes predicted from all three datasets in our stage one experiment where SNPs in chromosome X, Y and MT are excluded, predicted gene-gene interactions which have also been assigned with a biological function during our analysis based on GESA are selected for forming gene network. In this network, top 10 genes in terms of degree (i.e. number of directly interacting genes under our network) are listed in this table. For each gene G in this table, the number of directly interacting genes under our network and the number of these interactions which can be verified by the following 3 reference databases StringDB, BioGrid and RNAInter are listed in this table. Furthermore, the mean and variance of the number of intermediate interactors among the validated gene-gene interactions under the 3 reference networks are calculated and listed in this table. Genes associated to schizophrenia or other mental disorders are annotated with footnotes.

[1] Vrijenhoek et al., "Recurrent CNVs Disrupt Three Candidate Genes in Schizophrenia Patients."
[2] Verbrugghe et al., "Impact of the Reelin Signaling Cascade (Ligands--Receptors--Adaptor Complex) on Cognition in Schizophrenia."
[3] Gadelha et al., "Genome-Wide Investigation of Schizophrenia Associated Plasma Ndel1 Enzyme Activity."
[4] Raychaudhuri et al., "Accurately Assessing the Risk of Schizophrenia Conferred by Rare Copy-Number Variation Affecting Genes with Brain Function."
[5] Amato et al., "Schizophrenia and Vitamin D Related Genes Could Have Been Subject to Latitude-Driven Adaptation."
[6] Potkin et al., "A Genome-Wide Association Study of Schizophrenia Using Brain Activation as a Quantitative Phenotype."

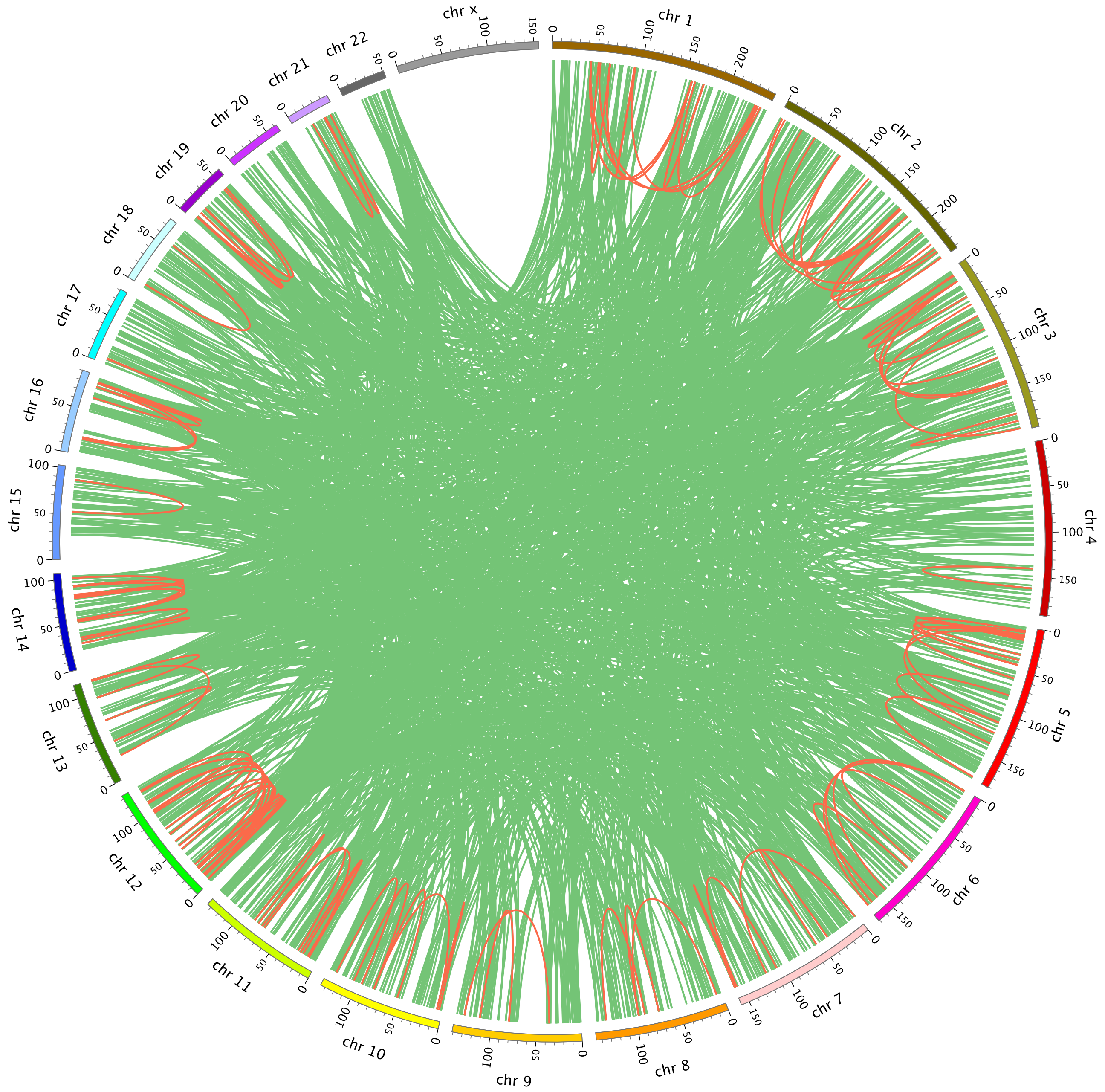

Supplementary Figure 1: This circos diagram visualizes the gene-gene interactions predicted from the top 10000 SNP-SNP interaction in terms of p-value found in our discovery dataset phs000021:phg000013 in our stage one experiment where SNPs in chromosome X, Y and MT are excluded.

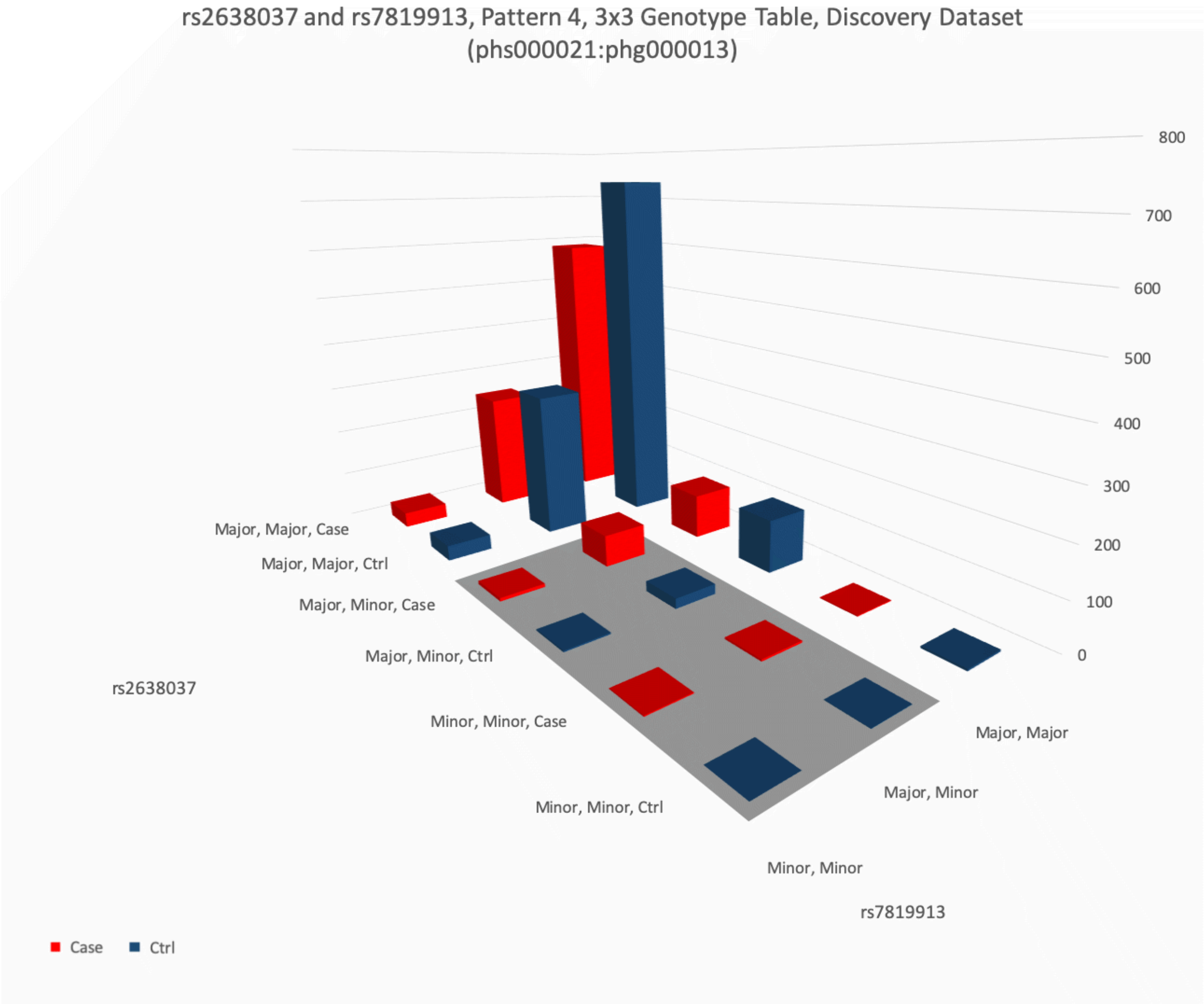

Supplementary Figure 2: This figure shows the distribution of genotypes of two SNPs rs2638037 and rs7819913. The height of the bar represents the number of samples of each of the nine combinations of the genotypes in these two SNPs. For each genotype combination, the bar in blue colour represents the number of healthy samples (controls) and the bar in red colour represents the number of patients (cases).

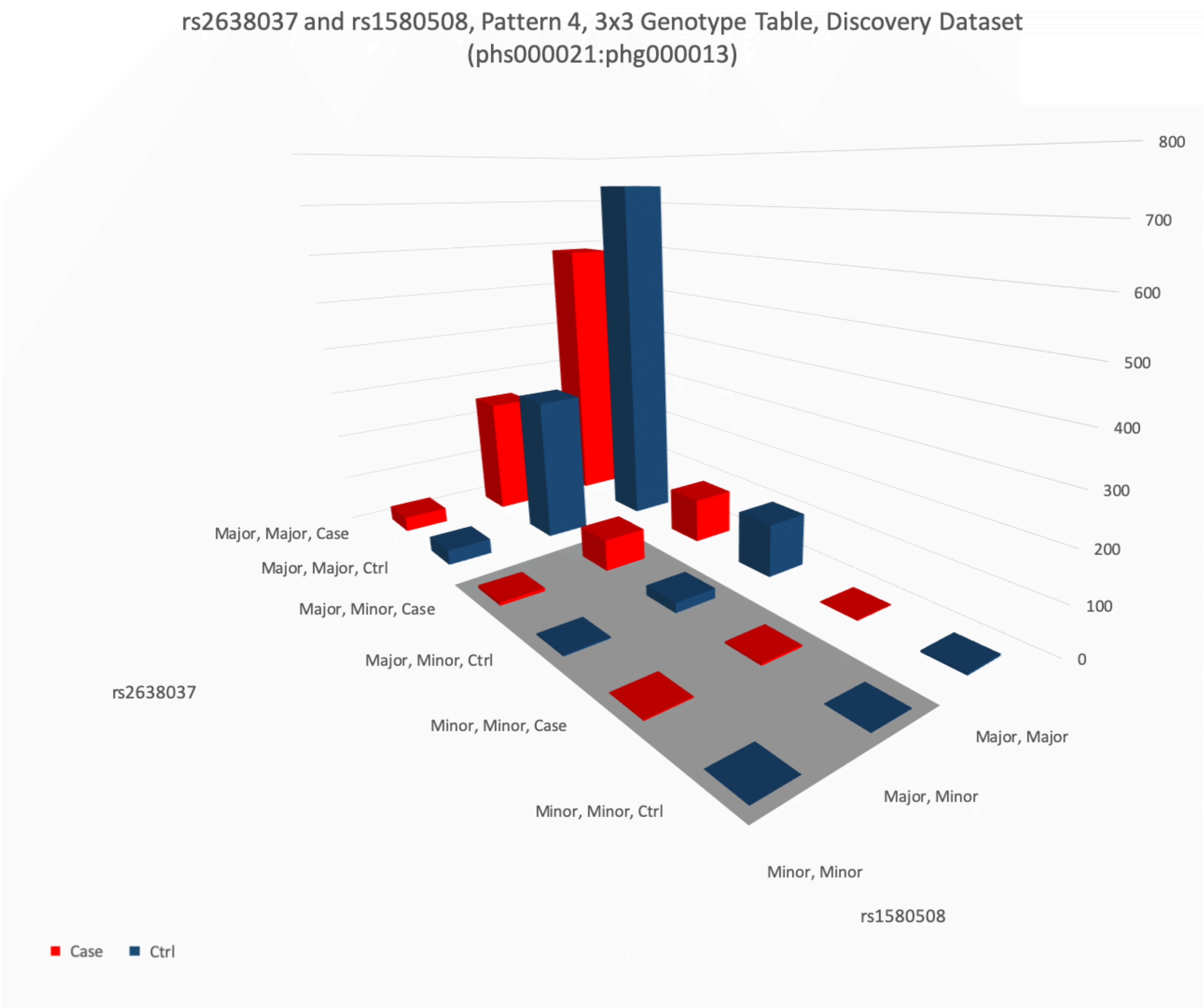

Supplementary Figure 3: This figure shows the distribution of genotypes of two SNPs rs2638037 and rs1580508. The height of the bar represents the number of samples of each of the nine combinations of the genotypes in these two SNPs. For each genotype combination, the bar in blue colour represents the number of healthy samples (controls) and the bar in red colour represents the number of patients (cases).

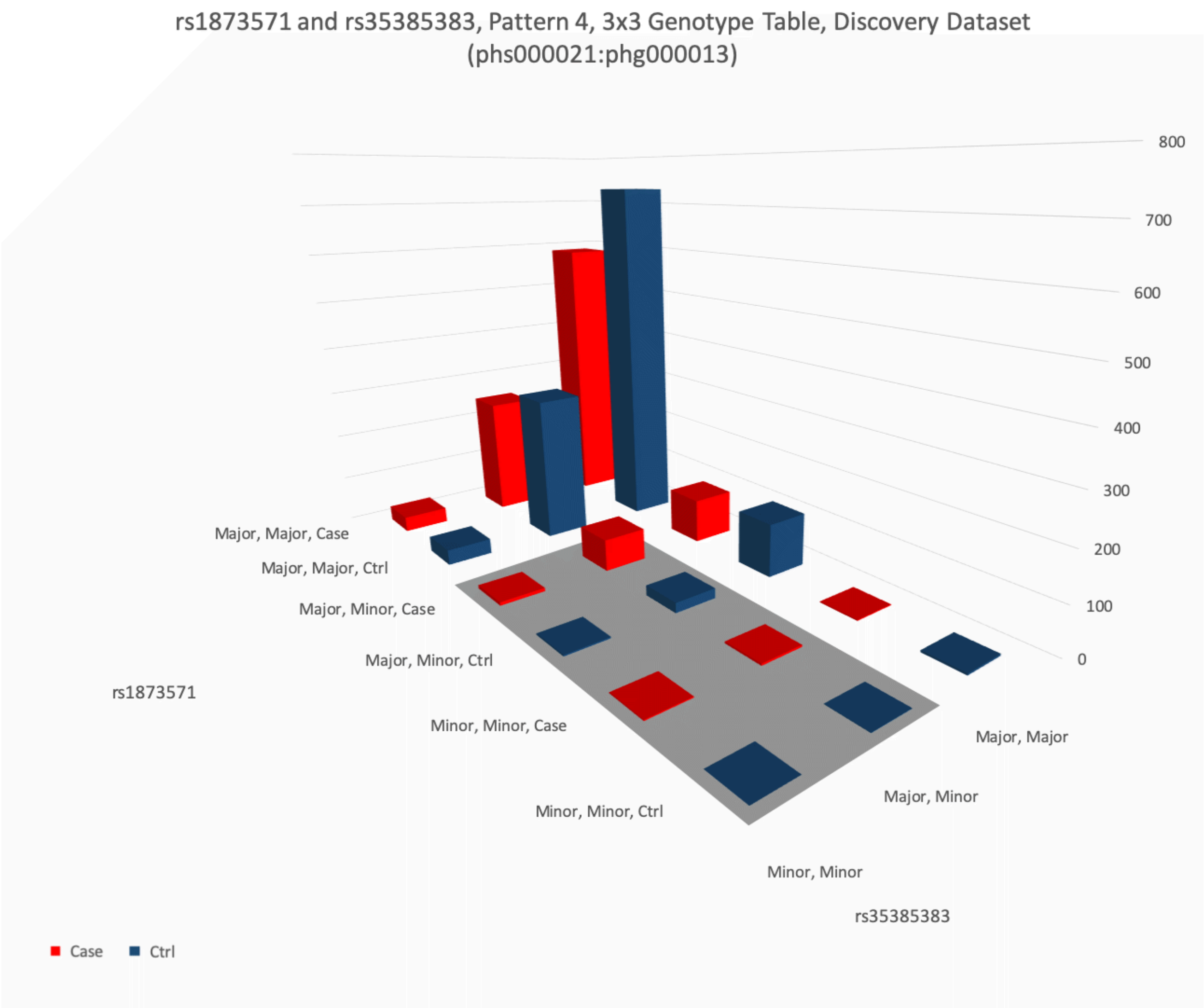

Supplementary Figure 4: This figure shows the distribution of genotypes of two SNPs rs1873571 and rs35385383. The height of the bar represents the number of samples of each of the nine combinations of the genotypes in these two SNPs. For each genotype combination, the bar in blue colour represents the number of healthy samples (controls) and the bar in red colour represents the number of patients (cases).

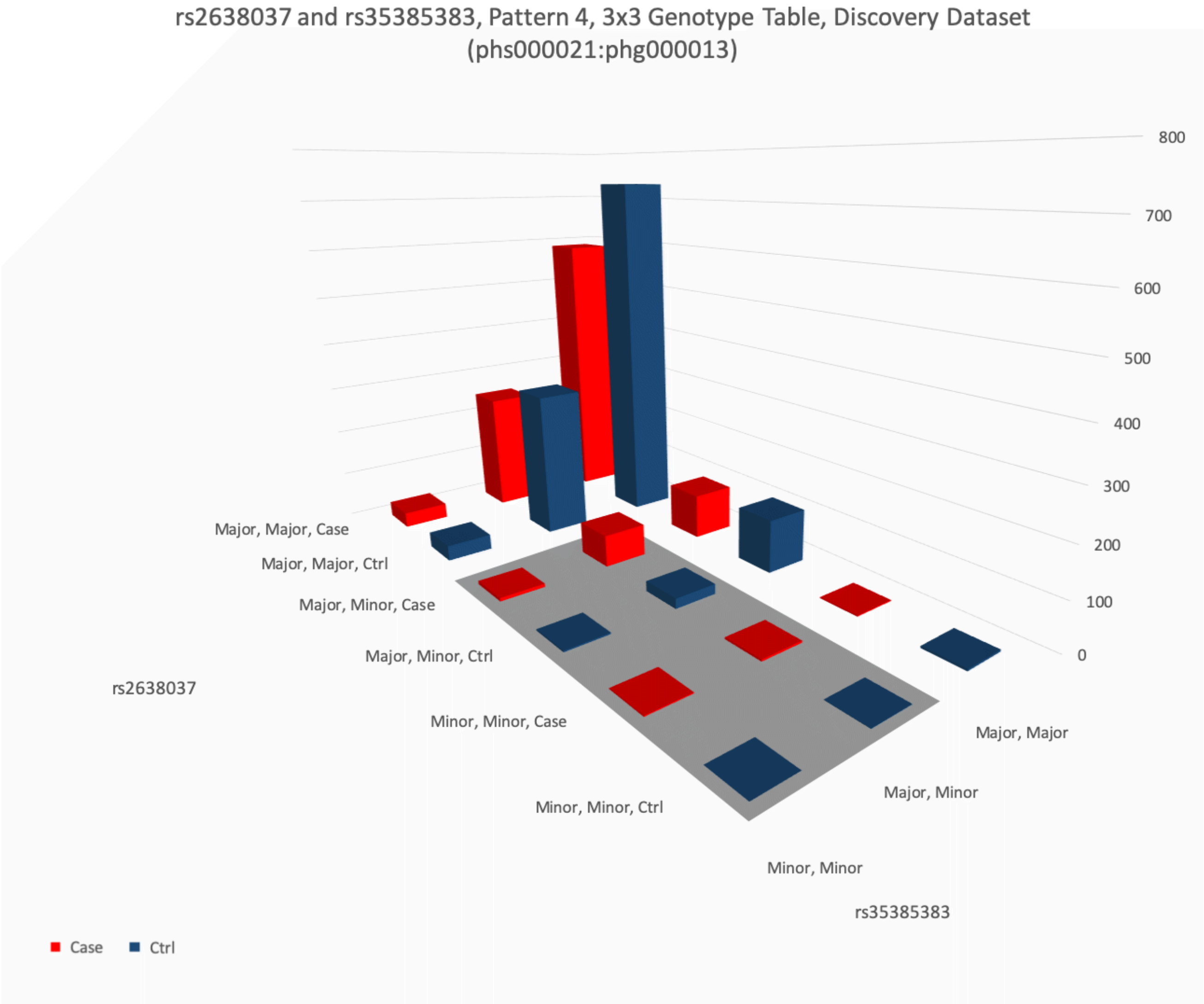

Supplementary Figure 5: This figure shows the distribution of genotypes of two SNPs rs2638037 and rs35385383. The height of the bar represents the number of samples of each of the nine combinations of the genotypes in these two SNPs. For each genotype combination, the bar in blue colour represents the number of healthy samples (controls) and the bar in red colour represents the number of patients (cases).

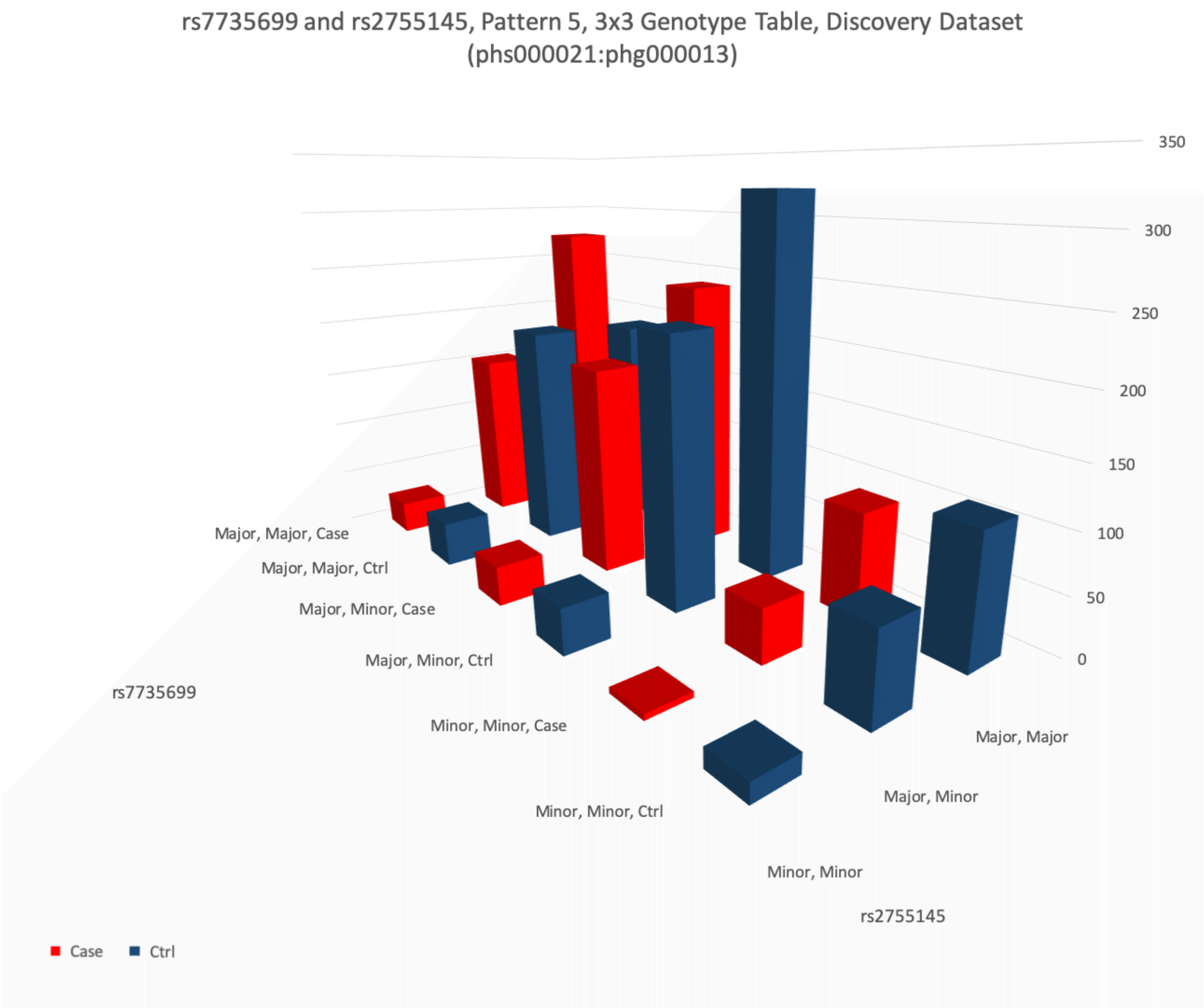

Supplementary Figure 6: This figure shows the distribution of genotypes of two SNPs rs7735699 and rs2755145. The height of the bar represents the number of samples of each of the nine combinations of the genotypes in these two SNPs. For each genotype combination, the bar in blue colour represents the number of healthy samples (controls) and the bar in red colour represents the number of patients (cases).

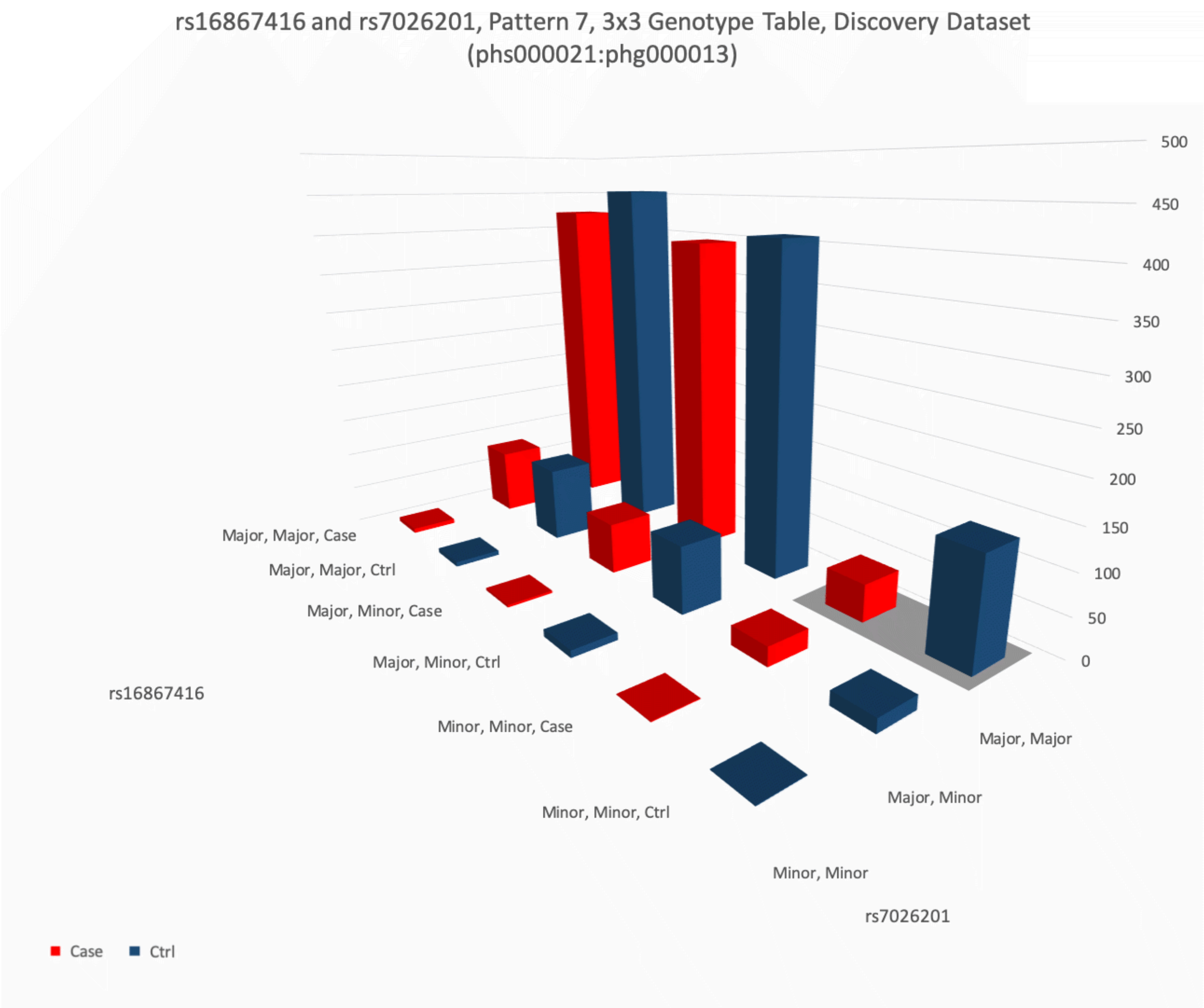

Supplementary Figure 7: This figure shows the distribution of genotypes of two SNPs rs16867416 and rs7026201. The height of the bar represents the number of samples of each of the nine combinations of the genotypes in these two SNPs. For each genotype combination, the bar in blue colour represents the number of healthy samples (controls) and the bar in red colour represents the number of patients (cases).

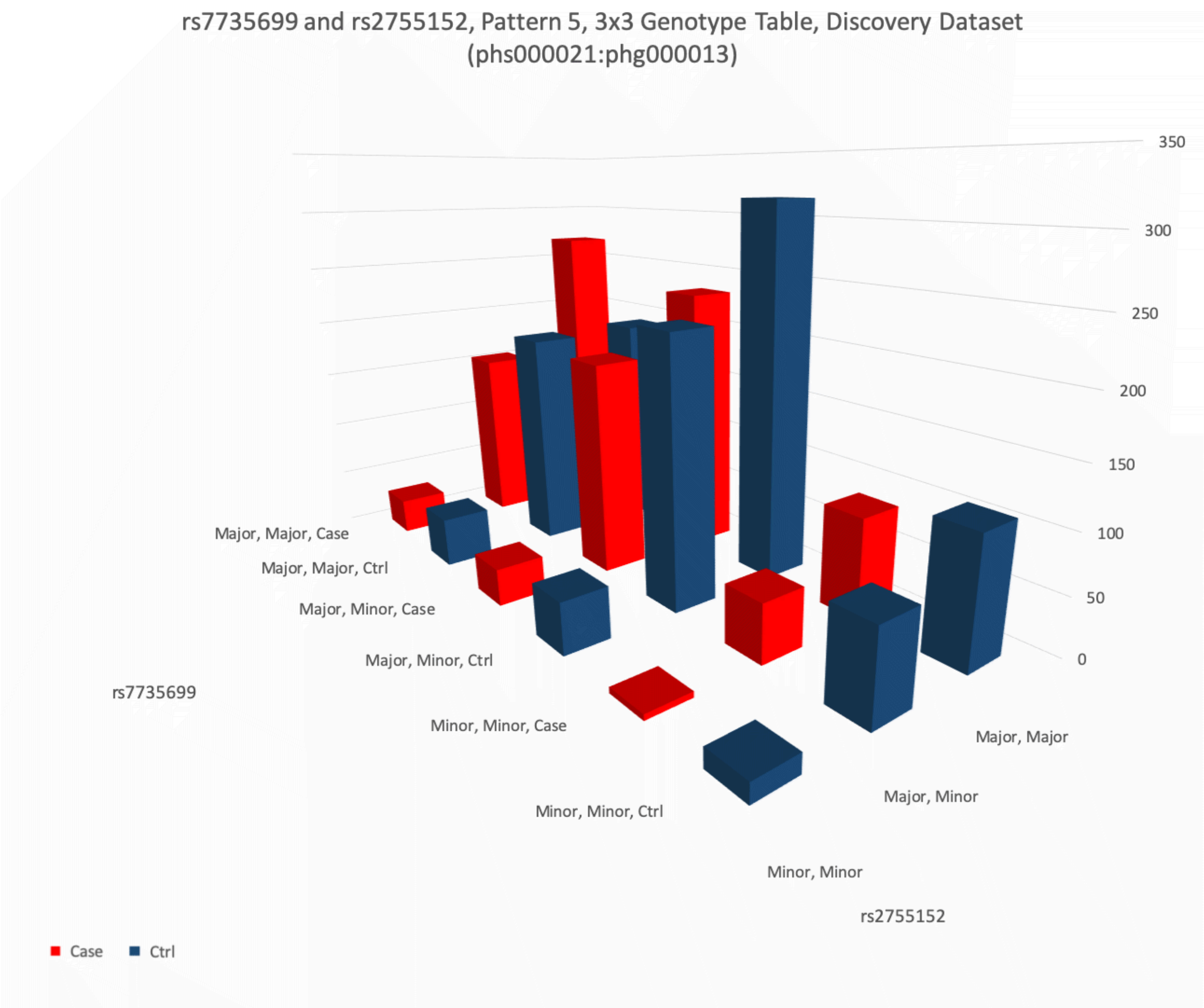

Supplementary Figure 8: This figure shows the distribution of genotypes of two SNPs rs7735699 and rs2755152. The height of the bar represents the number of samples of each of the nine combinations of the genotypes in these two SNPs. For each genotype combination, the bar in blue colour represents the number of healthy samples (controls) and the bar in red colour represents the number of patients (cases).

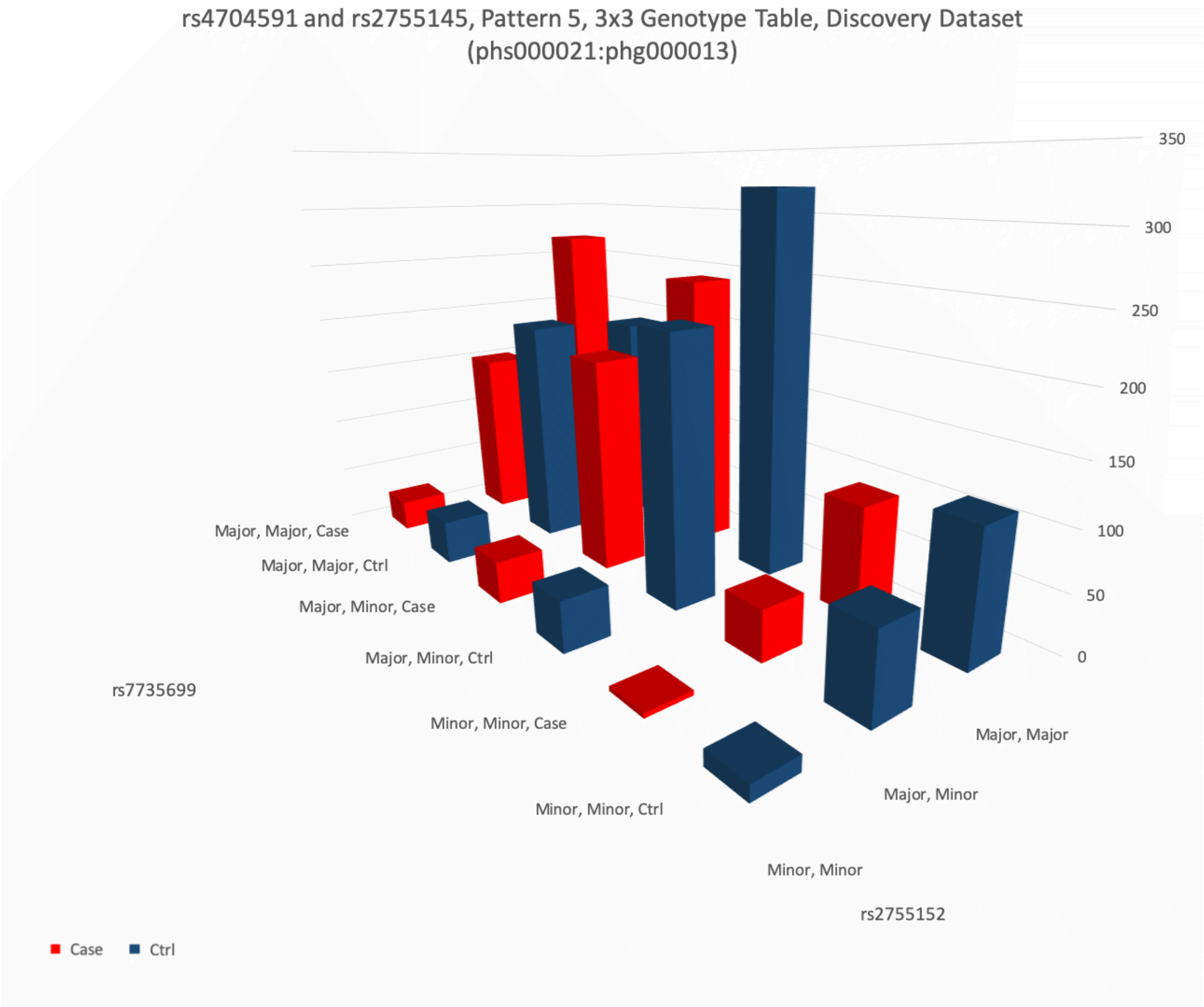

Supplementary Figure 9: This figure shows the distribution of genotypes of two SNPs rs4704591 and rs2755145. The height of the bar represents the number of samples of each of the nine combinations of the genotypes in these two SNPs. For each genotype combination, the bar in blue colour represents the number of healthy samples (controls) and the bar in red colour represents the number of patients (cases).

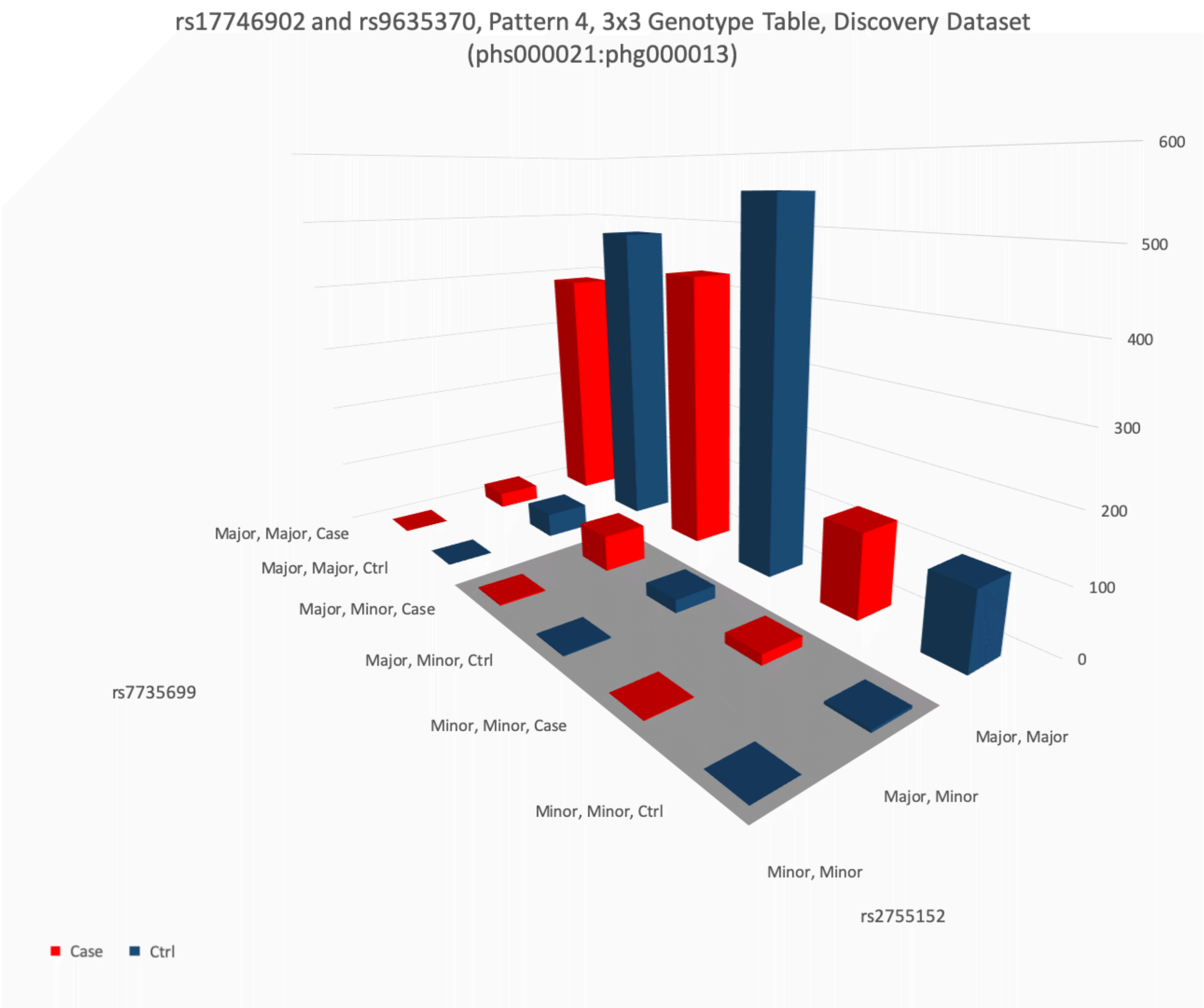

Supplementary Figure 10: This figure shows the distribution of genotypes of two SNPs rs17746902 and rs9635370. The height of the bar represents the number of samples of each of the nine combinations of the genotypes in these two SNPs. For each genotype combination, bars in blue colour represent the number of healthy samples (controls) and the bars in red colour represent the number of patients (cases).

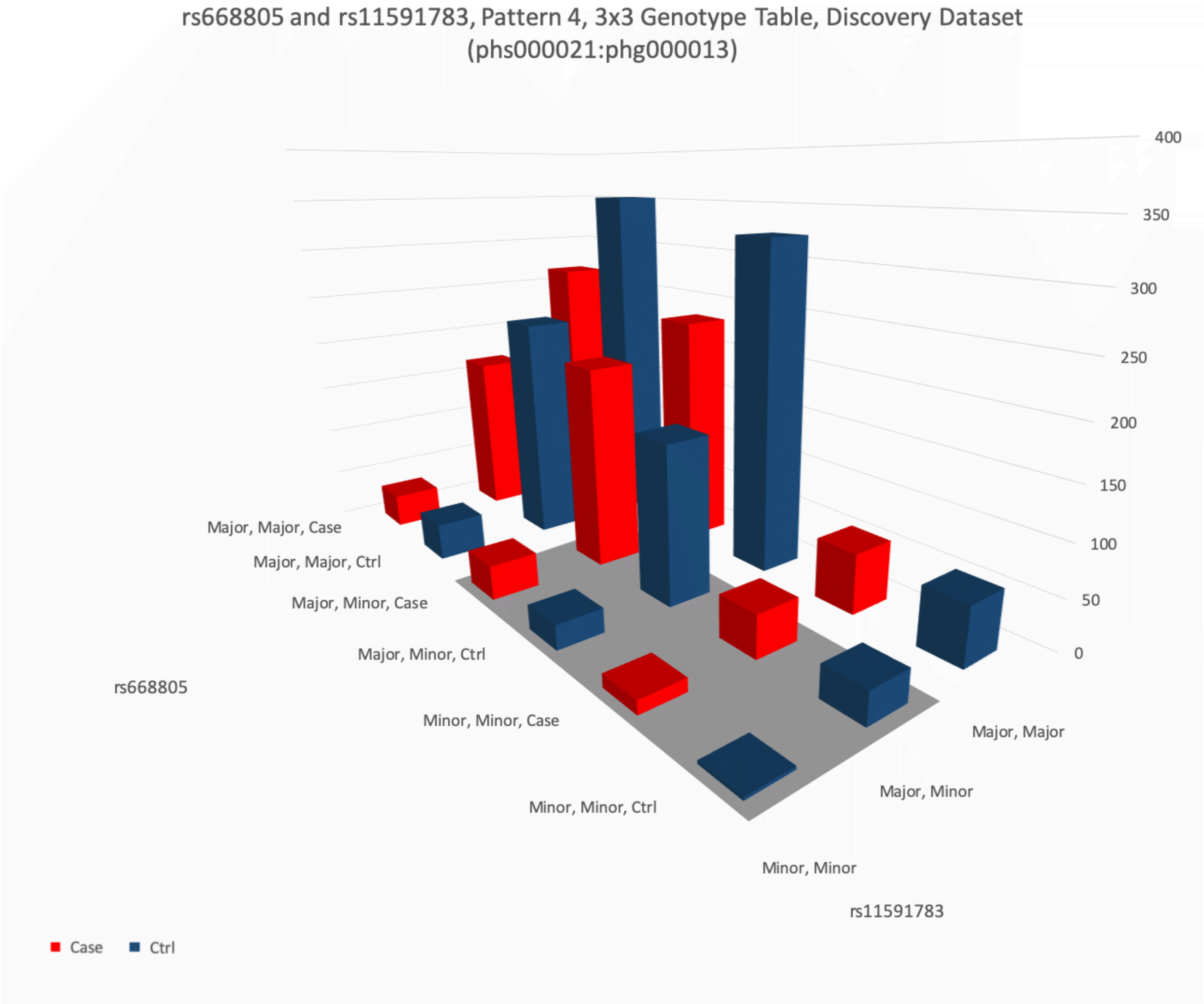

Supplementary Figure 11: This figure shows the distribution of genotypes of two SNPs rs668805 and rs11591783. The height of the bar represents the number of samples of each of the nine combinations of the genotypes in these two SNPs. For each genotype combination, bars in blue colour represent the number of healthy samples (controls) and the bars in red colour represent the number of patients (cases).

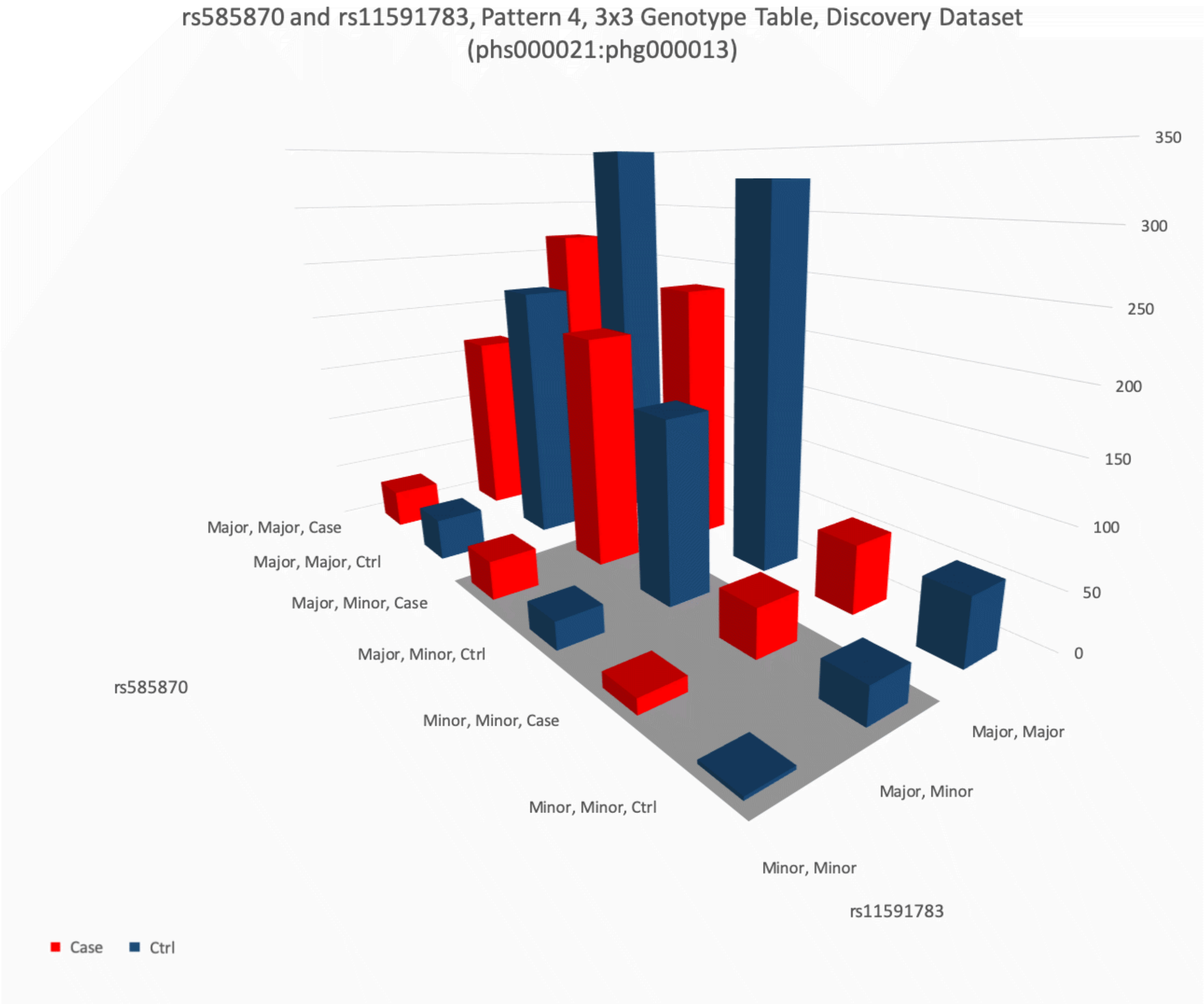

Supplementary Figure 12: This figure shows the distribution of genotypes of two SNPs rs585870 and rs11591783. The height of the bar represents the number of samples of each of the nine combinations of the genotypes in these two SNPs. For each genotype combination, bars in blue colour represent the number of healthy samples (controls) and the bars in red colour represent the number of patients (cases).

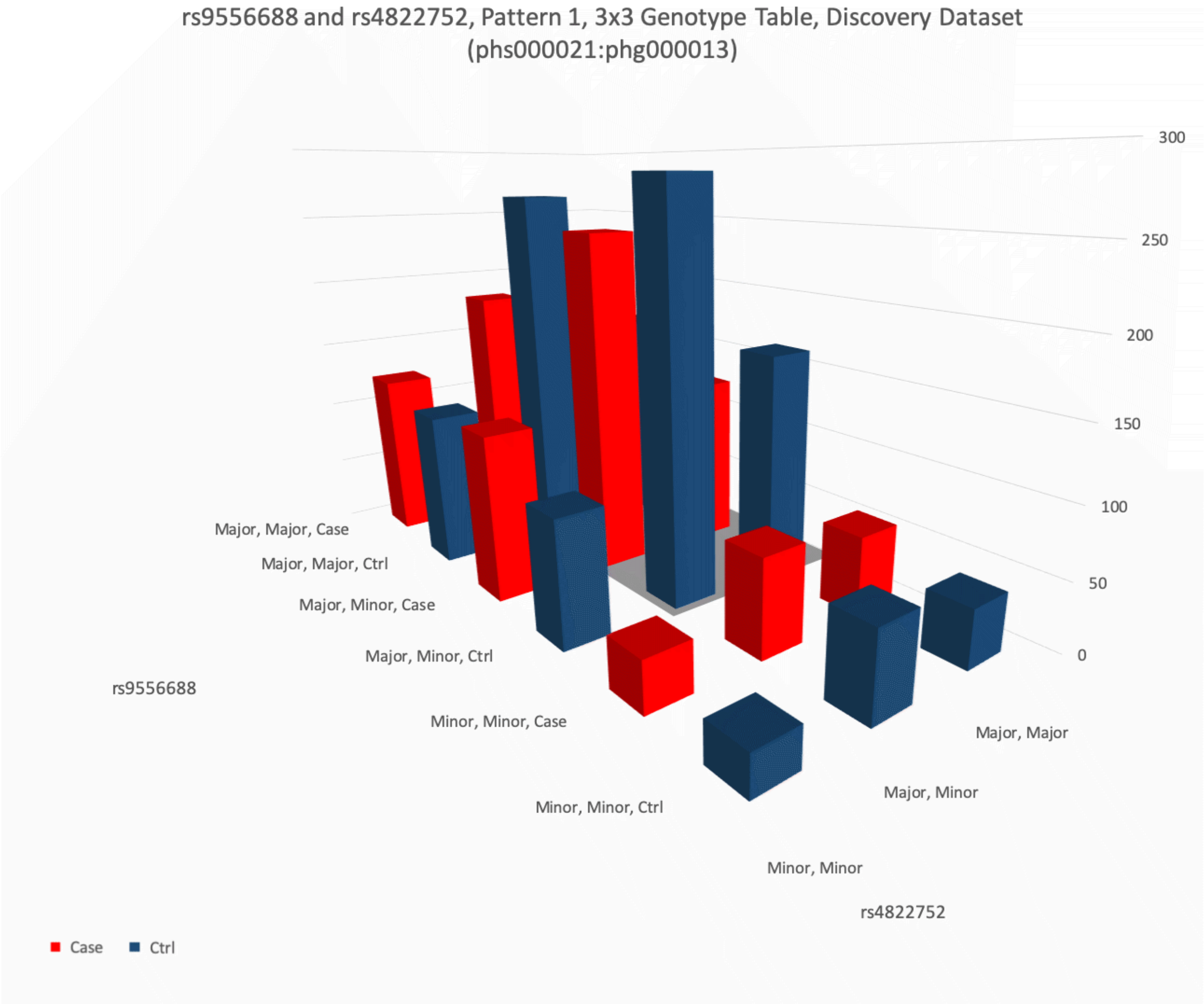

Supplementary Figure 13: This figure shows the distribution of genotypes of two SNPs rs9556688 and rs4822752. The height of the bar represents the number of samples of each of the nine combinations of the genotypes in these two SNPs. For each genotype combination, bars in blue colour represent the number of healthy samples (controls) and the bars in red colour represent the number of patients (cases).

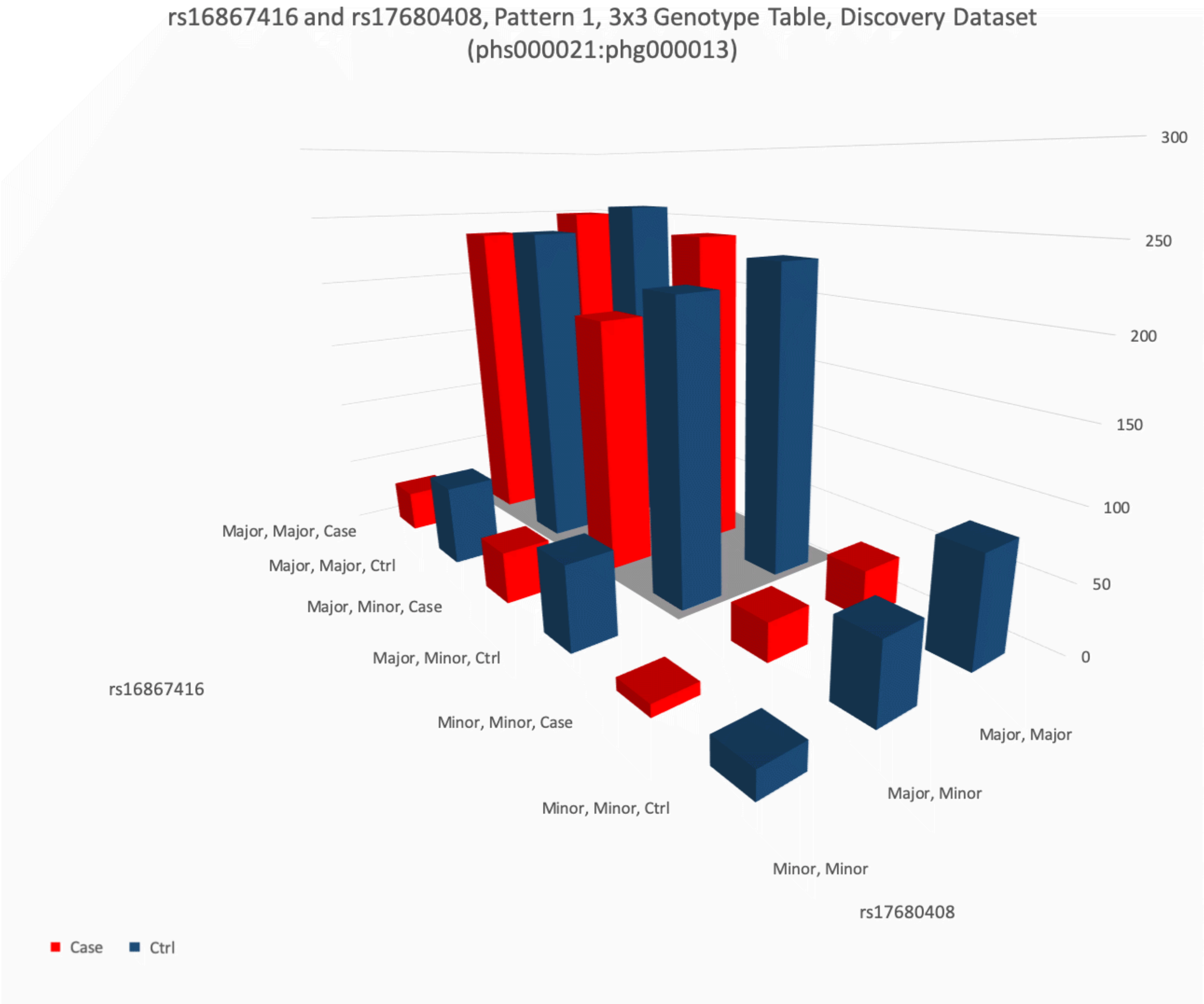

Supplementary Figure 14: This figure shows the distribution of genotypes of two SNPs rs16867416 and rs17680408. The height of the bar represents the number of samples of each of the nine combinations of the genotypes in these two SNPs. For each genotype combination, the bar in blue colour represents the number of healthy samples (controls) and the bar in red colour represents the number of patients (cases).
